# Δ133p53α, a natural p53 isoform, contributes to conditional reprogramming and long-term proliferation of primary epithelial cells

**DOI:** 10.1038/s41419-018-0767-7

**Published:** 2018-07-03

**Authors:** Abdul M. Mondal, Hua Zhou, Izumi Horikawa, Frank A. Suprynowicz, Guangzhao Li, Aleksandra Dakic, Bernard Rosenthal, Lin Ye, Curtis C. Harris, Richard Schlegel, Xuefeng Liu

**Affiliations:** 1 Center for Cell Reprograming, Department of Pathology, Georgetown University Medical Center, Georgrtown, WA 20057 USA; 2 Guizhou Medical University, Guiyang, Guizhou China; 3 Laboratory of Human Carcinogenesis, Center for Cancer Research, National Cancer Institute, National Institutes of Health, Bethesda, MD 20892 USA; 4 Shenzhen Eye Hospital, Shenzhen, Guangdong China; 5 Second Xianya Hospital (Adjunct Position), Zhongnan University, Changsha, Huna China; 6 Affiliated Cancer Hospital & Institute (Adjunct Position), Guangzhou Medical University, Guangzhou, Guangdong China

## Abstract

We previously developed the technique of conditional reprogramming (CR), which allows primary epithelial cells from fresh or cryopreserved specimens to be propagated long-term in vitro, while maintaining their genetic stability and differentiation potential. This method requires a combination of irradiated fibroblast feeder cells and a Rho-associated kinase (ROCK) inhibitor. In the present study, we demonstrate increased levels of full-length p53 and its natural isoform, Δ133p53α, in conditionally reprogrammed epithelial cells from primary prostate, foreskin, ectocervical, and mammary tissues. Increased Δ133p53α expression is critical for CR since cell proliferation is rapidly inhibited following siRNA knockdown of endogenous Δ133p53α. Importantly, overexpression of Δ133p53α consistently delays the onset of cellular senescence of primary cells when cultured under non-CR conditions in normal keratinocyte growth medium (KGM). More significantly, the combination of Δ133p53α overexpression and ROCK inhibitor, without feeder cells, enables primary epithelial cells to be propagated long-term in vitro. We also show that Δ133p53α overexpression induces hTERT expression and telomerase activity and that siRNA knockdown of hTERT causes rapid inhibition of cell proliferation, indicating a critical role of hTERT for mediating the effects of Δ133p53α. Altogether, these data demonstrate a functional and regulatory link between p53 pathways and hTERT expression during the conditional reprogramming of primary epithelial cells.

## Introduction

Primary human epithelial cells have a limited replicative lifespan in culture and their proliferation decreases rather rapidly (typically < 11 passages), leading to cellular senescence^1–3^. For decades, scientists have sought to develop methods for propagating normal and tumor primary cells efficiently and indefinitely for research in cancer biology and therapeutics^4,5^. Established methods for cellular immortalization involve the introduction of exogenous viral and/or cellular oncogene(s), such that these cell lines do not reflect a normal genotype^6–10^. Recently we established the technology of conditional reprogramming (CR), that enables normal and tumor primary epithelial cells to be propagated indefinitely in vitro while maintaining their original karyotype^11,12^. This methodology has opened up a new platform for basic and clinical research, with potential applications for regenerative and personalized medicine^13–15^.

The p53 tumor suppressor protein is a sequence-specific transcription factor that regulates cellular proliferation and apoptosis through the repression or activation of downstream target genes^16,17^. The absence of functional p53 leads to neoplastic transformation^18^. To date, 14 natural p53 isoforms (p53, p53β, p53γ, Δ40p53, Δ40p53β, Δ40p53γ, Δ133p53, Δ133p53β, Δ133p53γ, Δ160p53, Δ160p53β, Δ160p53γ, Δp53, and p53ψ) have been identified and many of them elicit distinct biological phenotypes^19–24^. While the functions of wild-type full-length p53 are well defined, the physiological role of various p53 isoforms in senescence, growth arrest and apoptosis are connected in a complex and often apparently conflicting manner. Previously, we showed that two p53 isoforms, Δ133p53α and p53β, potentially regulate cellular proliferation in human fibroblasts (MRC-5 and WI-38), lymphoid cells (CD8^+^ T lymphocytes) and astrocytes in vitro and in vivo^25–27^.

In the present study, we demonstrate that Δ133p53α regulates proliferation in conditionally reprogrammed epithelial cells isolated from prostates and foreskin tissues. Overexpression of Δ133p53α consistently delays cellular senescence and enables primary cells to be propagated in vitro indefinitely in the presence of a Rho-associated kinase (ROCK) inhibitor. The mechanism underlying Δ133p53α-extended replicative lifespan involves the upregulation of hTERT expression and its telomerase activity.

## Materials and methods

### Cell cultures and reagents

Neonatal foreskins (foreskin-1, foreskin-2), normal adult prostate tissues (prostate-1 and prostate-2), ectocervical and mammary tissues were collected from patients in accordance with Georgetown University Institutional Review Board (IRB) protocols^12,28,29^. Primary cells were isolated as described previously^11^. Briefly, samples were minced and digested with a mixture of dispase (Fisher Scientific) and collagenase (STEMCELL Technologies) and filtered through a 100-μm strainer to remove connective tissue. The isolated human foreskin keratinocytes (HFKs) and human prostate epithelial cells (HPECs) were cultured either in KGM [Keratinocyte-SFM supplemented with recombinant epidermal growth factor 1–53 (EGF 1–53) and bovine pituitary extract] (Gibco), or in CRC: F medium [3:1 (v/v) DMEM (Dulbecco's Modified Eagle Medium) (containing 10% (v/v) fetal bovine serum): F-12 nutrient mix] containing 0.125 ng/ml epidermal growth factor, 25 ng/ml hydrocortisone, 5 μg/ml insulin, 0.1 nM cholera toxin (Sigma-Aldrich), 10 μg/ml gentamicin, 250 ng/ml amphotericin B (Gibco) and 10 μM Y-27632 (Enzo Life Sciences)^11^ in the presence of irradiated Swiss 3T3-J2 fibroblasts. Where indicated, cells also were cultured in KGM containing 10 μM Y-27632 or in conditioned medium (CM) containing 10 μM Y-27632. CM was prepared from irradiated Swiss 3T3-J2 fibroblasts as described previously^11^. All cultures were maintained in a humidified incubator with 5% CO_2_ at 37 °C and passaged 1:4 (cultures without irradiated fibroblasts) or 1:8 (cultures with irradiated fibroblasts) when 80–90% confluent. Cell viability was determined by trypan blue exclusion before every passage. In addition to primary cells derived from tissue, MRC-5, WI-38, U2OS, HT1080, and 293T cell lines were from American Type Culture Collection. Population doublings were calculated as log_10_(final number of cells) – log_10_(initial number of cells)/log_10_2^25^. To quantify short-term proliferation ( < 8 days), cultures were monitored using the IncuCyte live-cell analysis system with IncuCyte ZOOM software (Essen BioScience).

### Separation of epithelial cells from irradiated fibroblasts

A two-step trypsin protocol was used to harvest epithelial cells from co-cultures with irradiated J2 fibroblasts^12^. Briefly, cultures were rinsed with Phosphate Buffered Saline (PBS) and incubated with 0.05% trypsin for 30–60 s at room temperature. J2 cells were removed by gentle tapping and aspiration. The epithelial cells were then rinsed with PBS, treated with trypsin for 3–5 min at 37 °C and detached from the flask by gentle tapping. Stop buffer (10% FBS in PBS) was added to neutralize the trypsin and the cell suspension was centrifuged for 5 min at 500 × *g*. Cell pellets were resuspended in medium for passaging or were washed with PBS at 4 °C and solubilized for analysis.

### Immunoblot analysis

Total cell lysates were prepared as previously described^25,26^. Briefly, cells were lysed in 1x RIPA buffer (Cell Signaling) containing a protease inhibitor cocktail (Sigma-Aldrich) and 0.1% SDS. Following sodium dodecyl sulfate polyacrylamide gel electrophoresis (Novex Tris-Glycine Gels, Invitrogen), samples were transferred to Polyvinylidene Fluoride (PVDF) membranes (Bio-Rad) and incubated with primary and secondary antibodies as listed below. Chemiluminescence was detected using western blotting Luminol Reagent (Santa Cruz Biotechnology) or SuperSignal West Dura Substrate (Pierce Biotechnology). Quantitative analysis of immunoblots was performed using ImageJ 1.40 software (http://rsb.info.nih.gov/ij/).

### Antibodies

The primary antibodies used were: DO-1 (1:1000; mouse monoclonal, Santa Cruz Biotechnology) for full-length p53; MAP4 (1:7500; rabbit polyclonal raised against a mixture of peptides MFCQLAKTC and FCQLAKTCP corresponding to the amino-terminus of human Δ133p53α, refs.^25,26,30^) for Δ133p53α; TLQi9 (1:5000; rabbit polyclonal raised against the peptide TLQDQTSFQKENC corresponding to the carboxyl-terminus of human p53β, TLQ40 or KJC8, refs. ^20,25^) for p53β, CM1 (1:1000; rabbit polyclonal, ref. ^24–26^) and GAPDH (0411) (1:1000; mouse monoclonal, Santa Cruz Biotechnology) for GAPDH. Secondary antibodies (horseradish peroxidase-conjugated goat anti-mouse IgG and goat anti-rabbit IgG) were obtained from Santa Cruz Biotechnology and used at a dilution of 1:5000.

### Preparation of lentiviral particles and transduction

Δ133p53α complementary DNA (cDNA)^25^ was cloned into the lentiviral vector pLoc-GFP-blasticidin (Open Biosystem). Lentiviral constructs, together with the Trans-Lentiviral GIPZ packaging system (Open Biosystem), were transfected into 293T/17 cells using Lipofectamine-2000 (Invitrogen). Viral particles were collected 48 h later and stored as aliquots at −80 °C. Vector control lentiviral particles (pLoc-RFP-GFP) were similarly prepared. Viral particles were titrated in HT1080 cells and showed > 10^6^ TU/ml. For transduction of epithelial cells, 50% confluent cultures were incubated with lentiviral particles for 6 h in the presence of 5 μg/ml polybrene (Santa Cruz Biotechnology). Subsequently, the polybrene-containing medium was replaced with fresh culture medium. After 2 days, the cells were re-plated at a dilution of 1:3–1:5, depending on the cell type and density. Two days later, cultures were selected for 10–12 days using 4–8 μg/ml blasticidin (Invitrogen).

### Quantitative reverse transcription PCR (qRT-PCR)

RNA was isolated from cell cultures using the RNeasy Plus Mini Kit (Qiagen). cDNA was generated using the SuperScript III First-Strand Synthesis System (Invitrogen). iQ SYBR Green Supermix (Bio-Rad #1708880) or SsoAdvanced Universal SYBR Green Supermix (Bio-Rad #1725270) was used for Δ133p53α qRT-PCR (forward primer 5′-ACT CTG TCT CCT TCC TCT TCC TAC AG-3′; reverse primer 5′-TGA GGA GGG GCC AGA CCA TC-3′)^25^, for full-length p53 (forward primer 5′-CTT CCC TGG ATT GGC AGC CA-3′; reverse primer 5′-CAT TCT GGG AGC TTC ATC TGG AC-3′), for p21 (forward primer 5′-ATG TCA GAA CCG GCT GGG GA-3′; reverse primer 5′-GCC GTT TTC GAC CCT GAG AG-3′), for E2F1 (forward primer 5′-TGC TCG ACT CCT CGC AGA TC-3′; reverse primer 5′-AGG AAG CGC TTG GTG GTC AG-3′), for PUMA (forward primer 5′-GAC TCC TGC CCT TAC CCA G-3′; reverse primer 5′-ATG GTG CAG AGA AAG TCC C-3′), for BAX (forward primer 5′-AAG AAG CTG AGC GAG TGT-3′; reverse primer 5′-GGA GGA AGT CCA ATG TC-3′) and for NOXA (forward primer 5′-CGG AGA TGC CTG GGA AGA AG-3′; reverse primer 5′-AGG AGT CCC CTC ATG CAA GT-3′). The internal control was β2-microglobulin (forward primer 5′-GGA CTG GTC TTT CTA TCT CTT GT-3′; reverse primer 5′-ACC TCC ATG ATG CTG CTT AC-3′). iQ Supermix (Bio-Rad #1708862) was used for Taqman qRT-PCR to quantify hTERT mRNA expression using the following primers and probe: forward primer 5′-TGA CAC CTC ACC TCA CCC AC‑3′; reverse primer 5′-CAC TGT CTT CCG CAA GTT CAC‑3′; probe 5′-ACC CTG GTC CGA GGT GTC CC-3′. Normalized RNA expression was calculated using the ΔΔCt method according to the supplier’s protocol (Bio-Rad CFX Manager software).

### Small interfering RNA (siRNA) knockdown

A stealth siRNA duplex oligoribonucleotide targeting Δ133p53α mRNA (Δ133-si#1; 5′-UGU UCA CUU GUG CCC UGA CUU UCA A‑3′), its scrambled control and a standard siRNA duplex oligoribonucleotide targeting Δ133p53α mRNA (Δ133-si#2; 5′-CUU GUG CCC UGA CUU UCA A[dT][dT]‑3′) were purchased from Invitrogen. Both Δ133-si#1 and Δ133-si#2 were designed to target regions that are present in Δ133p53α mRNA as a 5′-UTR but spliced out of full-length p53α mRNA as intron 4^25^. For hTERT knockdown, the following siRNAs were purchased from Dharmacon: siGENOME hTERT SMARTpool (5′-GGU AUG CCG UGG UCC AGA A-3′, 5′-CCA CGU CUC UAC CUU GAC A-3′, 5′-UCA CGG AGA CCA CGU UUC A-3′ and 5′-GCG UGG UGA ACU UGC GGA A-3′) and siGENOME non-targeting siRNA (5′-UAG CGA CUA AAC ACA UCA A-3′). HFKs and HPECs were transfected with siRNAs at a final concentration of 12 nM for Δ133p53α knockdown and 25 nM for hTERT knockdown using Lipofectamine RNAiMAX or Lipofectamine 3000 transfection reagents (Invitrogen) according to the manufacturer’s protocols.

### Real-time quantitative TRAP assay for telomerase activity

HFKs and HPECs transduced with Δ133p53α or vector control were grown to 80% confluence in 25 mm^2^ flasks, harvested by trypsin treatment, washed in cold PBS, and transferred to micro-centrifuge tubes. Cell pellets were lysed for 30 min at 4 °C in 200 μl of TRAP buffer (0.5% Chaps, 10 mM Tris-HCl pH 7.5, 1 mM MgCl_2_, 1 mM EGTA, 5 mM β-mercaptoethanol, 10% glycerol and 0.1 mM 4-(2-aminoethyl)-benzenesulfonyl fluoride hydrochloride). Lysates were centrifuged at 14,000 × *g* for 5 min at 4 °C, supernatants were transferred to new tubes, and protein concentrations were determined using the Pierce 660 nm Protein Assay Reagent (Thermo Fisher). A quantitative TRAP assay was performed as described^31–34^, with some modifications. Briefly, 1.0 μg of lysate protein was incubated for 60 min at 33 °C in a 40 μl reaction volume containing 1x PCR buffer (20 mM Tris-HCl pH 8.4, 50 mM KCl), 1.5 mM MgCl_2_, 0.5 μM telomerase substrate primer (5′-AAT CCG TCG AGC AGA GTT-3′), 125 μM of each deoxynucleotide triphosphate (dATP, dTTP, dGTP, and dCTP) and 0.5 μg of T4 gene protein (Roche Applied Science). Telomerase was inactivated by heating at 95 °C for 10 min. In the second step of the assay, SYBR Green qRT-PCR was performed to quantitate the number of substrate molecules to which telomere repeats had been added. Each 25-μl reaction contained 300 nM of the above telomerase substrate primer and ACX primer (5′-GCG CGG CTT ACC CTT ACC CTT ACC CTA ACC-3′), and 1.0 μl of product from the first step of the assay. A standard curve was generated for the quantitative TRAP assay using serially diluted HEK 293T cell extracts. All samples were assayed in triplicate. This assay is linear over at least a 500-fold range (0.008–4 μg of HEK 293T protein input).

### Senescence-associated β-galactosidase (SA-β-gal) assay

KGM cells of HFKs at passage 4 (early passage) or passage 10 (late passage), CRC cells of HPECs after three passages in CRC or in CRC without feeders, and control or Δ133p53α-overexpressing HFK cells at passage 6 in KGM were examined using Senescence β-Galactosidase Staining Kit (Cell Signaling) per the manufacturer’s instructions.

### Statistics

Statistical analyses were carried out using a two-tailed Student’s *t*-test for paired and unpaired samples as appropriate. A *P*-value < 0.05 was considered significant.

## Results

### CR cells exhibit increased expression of full-length p53 and Δ133p53α

**Fig. 1:**
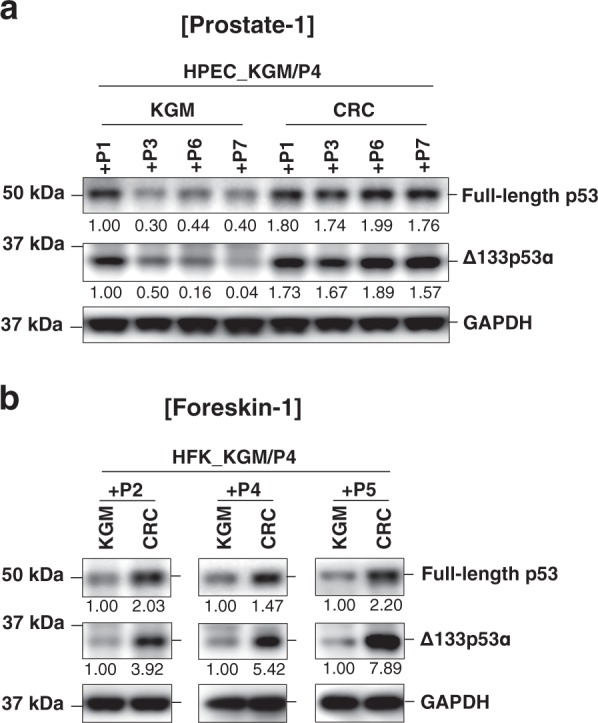
Increased expression of full-length p53 and Δ133p53α in CR cells. **a** Immunoblot analysis of full-length p53 and Δ133p53α proteins in HPECs (prostate-1) cultured in keratinocyte growth medium (KGM) or CR culture (CRC) for 1–7 passages after four passages in KGM (+P1 through +P7). GAPDH was used as a loading control for quantification. Normalized densitometric values for expression levels are indicated below each lane relative to +P1 KGM cells (defined as 1.0). **b** Immunoblot analysis of full-length p53 and Δ133p53α proteins in HFKs (foreskin-1) cultured in KGM or CRC for 2–5 passages after four passages in KGM (+P2 through +P5), using GAPDH as the loading control. Normalized densitometric values for expression levels are indicated below each lane relative to +P2 KGM cells (defined as 1.0). Immunoblot analysis of Δ133p53α proteins in HMEC and HEC cells cultured in KGM or CRC are shown in Supplementary Information Fig. S2. Uncropped blot images are shown in Supplementary Information Figs. S20–23

Since full-length p53 and Δ133p53α play important roles in the regulation of cellular senescence^25,26,35^ in several non-epithelial cell types, we measured their expression in primary HPECs grown in two culture conditions: (i) traditional keratinocyte growth medium (KGM), which does not support long-term cell proliferation, and (ii) conditional reprogramming culture (CRC), which mediates the long-term growth of many epithelial and non-epithelial cell types^11,12^. Both full-length p53 and Δ133p53α were consistently at higher levels in the prostate CRC cells compared to the cells cultured in KGM (Fig. 1a and Supplementary Information Fig. S1a). Increased levels of full-length p53 and Δ133p53α were also observed in CRC cells of primary HFKs (Fig. 1b and Supplementary Information Fig. S1b). In addition, the increased levels of Δ133p53α protein isoform in the CRC cells were consistent in other cell types, primary human ectocervical cells (HECs) and human mammary epithelial cells (HMECs) (Supplementary Information Fig. S2). We examined another p53 isoform, p53β, which plays role in p53-mediated senescence and apoptosis^20^, was undetectable in these cell types; even at late passage in KGM where cells stopped dividing (Supplementary Information Fig. S3). Therefore, p53β isoform was ruled out as a significant regulator of CR in this study.

### Increased Δ133p53α expression correlates with CR rescue of late-passage KGM cultures

**Fig. 2:**
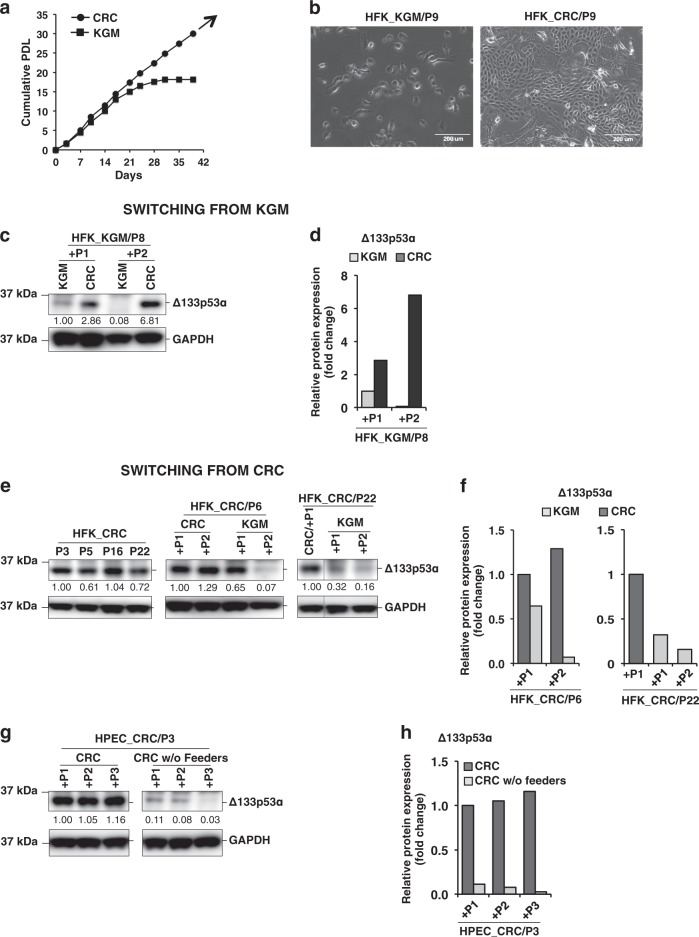
Increased Δ133p53α expression correlates with cell proliferation. **a** Freshly isolated HFKs were cultured in KGM or CRC until the KGM cells stopped proliferating. Cumulative population doubling levels (PDL) were plotted vs. time (days). **b** Representative images of the HFKs at passage 9 in KGM or CRC. Scale bars: 200 μm. **c** Late-passage HFKs (P8) cultured in KGM were shifted to CRC (or maintained in KGM) for an additional two passages (+P1 and +P2). The expression of Δ133p53α in these cultures was determined by immunoblot analysis. GAPDH was the loading control. Normalized densitometric values are indicated below each lane. **d** Bar graph of Δ133p53α expression in the cultures shown in **c**. **e** HFKs cultured in CRC for 6 or 22 passages were transferred to KGM (or maintained in CRC) for an additional two passages (+P1 and +P2). Immunoblot analysis was used to quantify Δ133p53α expression. Δ133p53α levels were consistently high in CRC (P3 through P22), however, expression dramatically decreased when the cells were switched to KGM. GAPDH was the loading control. Normalized densitometric values are indicated below each lane. **f** Bar graph of Δ133p53α expression in the middle and right panels shown in **e**. **g** Δ133p53α levels in HPECs initially cultured in CRC for three passages (HPEC_CRC/P3) before transferring to CRC with or without irradiated feeder cells for an additional three passages (+P1,+P2, and +P3). GAPDH was the loading control. Normalized densitometric values are indicated below each lane. **h** Bar graph of the data shown in **g**. Uncropped blot images are shown in Supplementary Information Figs. S26–28

As shown previously^11,12^, normal human epithelial cells have a limited replicative lifespan in standard KGM culture, but can be propagated indefinitely in CR culture (Fig. 2a, b). However, late-passage KGM cells regain the capacity to proliferate if the culture conditions switched to CRC (Supplementary Information Fig. S4). Renewed proliferation was found to correlate with increased Δ133p53α expression when passage 8 KGM cells of HFK (close to senescence; see Supplementary Information Fig. S5) were placed in CR culture for 1 or 2 passages (Fig. 2c, d). However, though there was initial increase of full-length p53, the rescued CRC cells showed lower levels of full-length p53 compared to their KGM counterparts (Supplementary Information Fig. S6). The increased p53 levels associated with enhanced proliferation in CR were not consistent when rescued at the later stage of their replicative lifespan. On the other hand, we observed consistent and abundant levels of Δ133p53α in the immediately rescued CRC cells and that prompted us to pursue investigating mainly the role of Δ133p53α isoform in CR. Δ133p53α expression in CR HFKs remained at a high level for at least 22 passages, but decreased rapidly (1–2 passages) and dramatically (up to 15-fold) when transitioned to KGM culture (Fig. 2e, f). The decrease in Δ133p53α expression coincided with a rapid (1–2 passages) cessation of proliferation (Supplementary Information Fig. S7a, b). The correlation between proliferation and Δ133p53α levels was further demonstrated by removing irradiated mouse 3T3-J2 feeder cells from CR culture of HPECs. Following removal of the feeders, the HPECs stopped proliferating and became senescent within three passages (Supplementary Information Fig. S8a, b). The decrease in proliferative capacity coincided with decreased Δ133p53α expression (Fig. 2g, h). Altogether, these results show that Δ133p53α expression correlates with the proliferative capacity of primary epithelial cells in vitro, which is dependent upon feeder cells.

### Knockdown of endogenous Δ133p53 inhibits cell proliferation

**Fig. 3:**
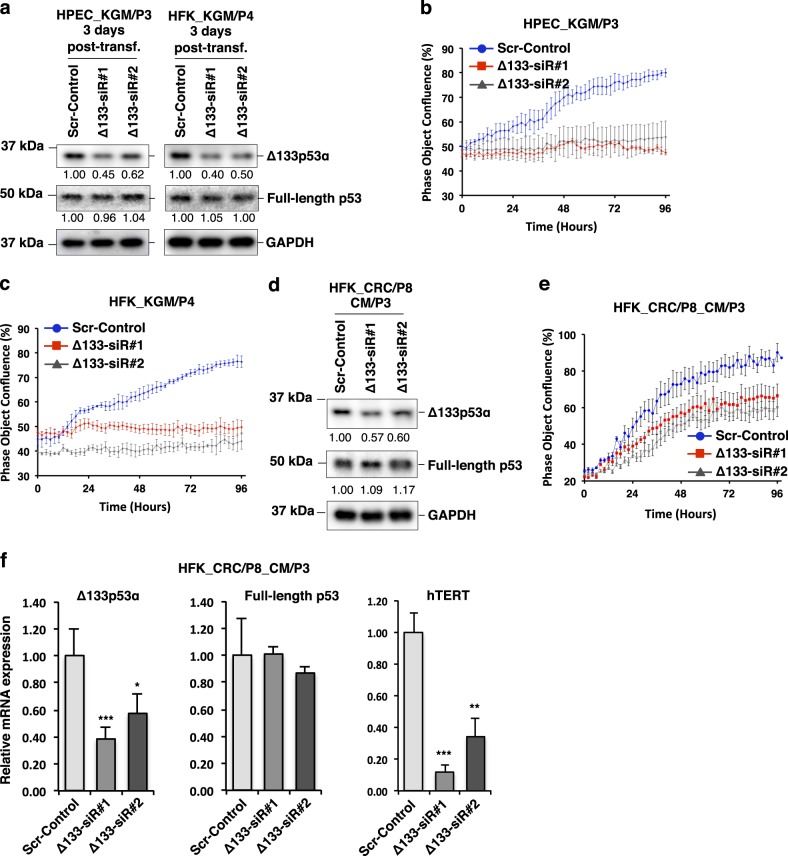
Knockdown of endogenous Δ133p53α inhibits cell proliferation in KGM and CR cultures. Two independent siRNAs (Δ133-siR#1 and Δ133-siR#2) were designed to target sequences present in Δ133p53α mRNA as 5′-UTRs that are spliced out of full-length p53 mRNA. Transfection was performed in KGM using early-passage HPECs (P3) and HFKs (P4). The control siRNA (Scr-Control) was a scrambled sequence of Δ133-siR#1. **a** Immunoblot analysis of Δ133p53α and full-length p53 proteins at 3 days post-transfection. GAPDH was used as a loading control. Normalized densitometric values for expression levels are indicated below each lane relative to Scr-Control cells (defined as 1.0). Representative images of the transfected HFKs are shown in Supplementary Information Fig. S9a, b. **b** Proliferation of the transfected HPECs and **c** HFKs were monitored for 4 days using IncuCyte. Data are mean ± S.D. from triplicate wells. **d** Immunoblot analysis for Δ133p53α and full-length p53 proteins at 3 days post-transfection of the HFKs with Scr-Control, Δ133-siR#1 and Δ133-siR#2 siRNA in conditioned media (CM). These HFKs were initially cultured in CRC for eight passages (HFK_CRC/P8) and then cultured in CM for another three passages (CM/P3) before transfection. GAPDH was used as a loading control. Normalized densitometric values are indicated below each lane. **e** Proliferation of the transfected HFKs in **d** were monitored for 4 days post-transfection using IncuCyte. Data are the mean ± S.D. from triplicate wells. Representative images of the transfected HFKs are shown in Supplementary Information Fig. S10. **f** Expression of Δ133p53, full-length p53 and hTERT mRNA in the transfected HFKs in **d** were measured by qRT-PCR at 3 days post-transfection in conditioned medium (CM). β2-microglobulin mRNA was used for normalization. Data are mean ± S.D. from three independent experiments. *P < 0.05; **P < 0.01; ***P < 0.001. Uncropped blot images are shown in Supplementary Information Figs. S29–30

To investigate the importance of Δ133p53α for the proliferation of CR cells, we first transfected early-passage (P3–4) HFKs and HPECs in KGM culture with two siRNAs (Δ133p53α-siR#1 and Δ133p53α-siR#2) previously used to knockdown Δ133p53α mRNA in human fibroblasts and lymphocytes^25,26^. As shown (Fig. 3a), Δ133p53α-siR#1 reduced Δ133p53α protein levels by 60% and Δ133p53α-siR#2 by 40%, without affecting full-length p53 levels. We then continuously monitored the proliferation of both knockdown and control cells (transfected with scrambled sequence siRNA) in KGM using IncuCyte technology (see Methods) for 4 days after transfection. HFKs and HPECs transfected with Δ133p53α-siRNAs underwent immediate growth arrest, whereas cells transfected with the control siRNA continued to proliferate at the same rate as non-transfected cells (Fig. 3b, c and Supplementary Information Fig. S9a, b). We next investigated the importance of Δ133p53α for the proliferation of CRC cells by similarly knocking down endogenous Δ133p53α expression in the conditionally reprogrammed HFKs (Fig. 3d, f). The inhibition of cell proliferation was accompanied by loss of endogenous Δ133p53α protein in the CR cells (Fig. 3d, e and Supplementary Information Fig. S10). As the conditionally reprogrammed cells showed increased basal levels of Δ133p53α, the siRNA-knockdown effects for growth inhibition took a little longer and were less efficient compared to the KGM cells where basal levels of Δ133p53α were relatively low (Fig. 3c, e). However, the siRNAs used to target the Δ133p53 mRNA also knockdown another p53 protein isoform Δ160p53α at the same time^24^. Therefore, we further investigated the Δ133p53 mRNA knockdown cells using CM1 antibody that reacts with both the Δ133p53α and Δ160p53α protein isoforms^24^. None of the control or Δ133p53 siRNA transfected cells showed any detectable Δ160p53α protein expression (Supplementary Information Fig. S11). Interestingly, we also noticed that knockdown of Δ133p53α isoform was associated with the loss of hTERT mRNA expression without affecting full-length p53 expression (Fig. 3f). These results suggest that Δ133p53α expression is necessary for the increased proliferation of CR epithelial cells and perhaps by upregulating hTERT expression.

### Overexpression of Δ133p53α extends the replicative lifespan of primary epithelial cells in KGM

**Fig. 4:**
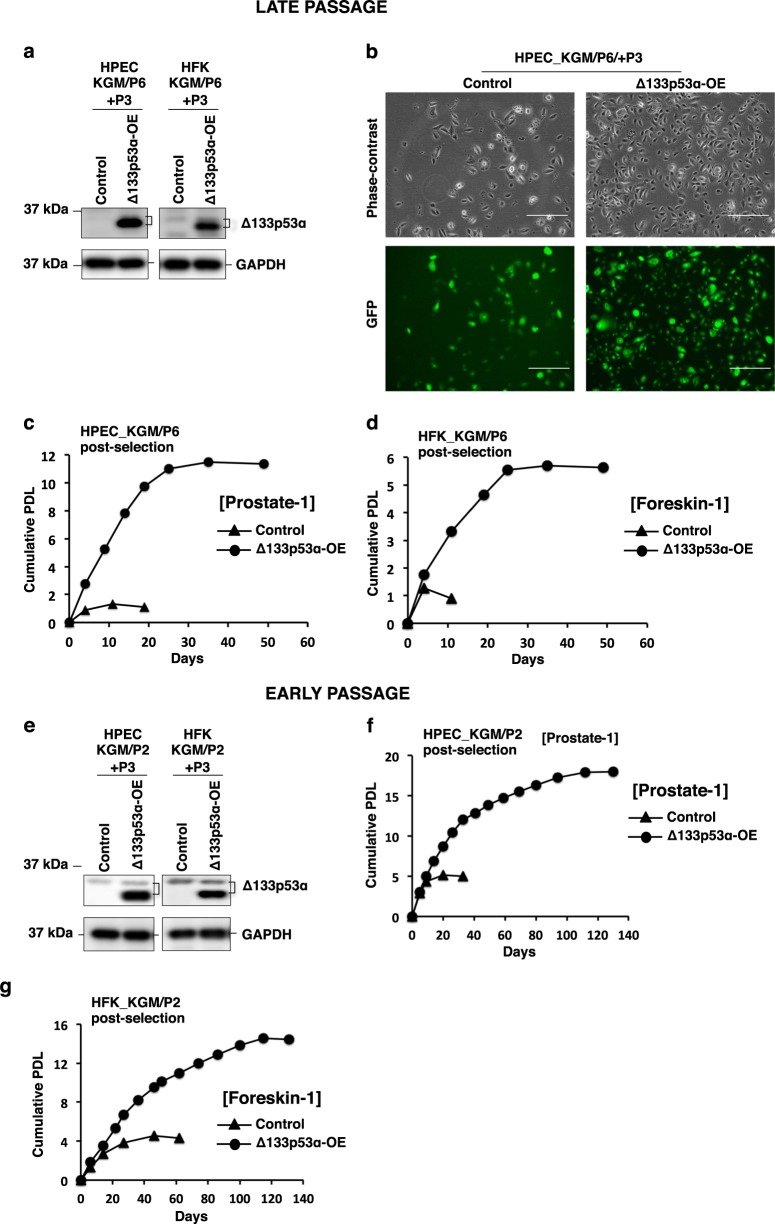
Overexpression of Δ133p53α extends the replicative lifespan. Late-passage (P6) KGM cultures of HPECs and HFKs were transduced with lentivirus containing an empty GFP-tagged vector (control) or GFP-tagged Δ133p53α to induce Δ133p53α overexpression (Δ133p53α-OE) and selected with blasticidin for three passages (+P3). **a** Immunoblots showing Δ133p53α overexpression (lower band). Slightly slower migrating band represents their endogenous levels. GAPDH was used as a loading control. **b** Representative images of control and Δ133p53α-OE HPECs three passages after selection (HPEC_KGM/P6/+P3). Upper panel: phase-contrast microscopy. Lower panel: GFP fluorescence microscopy. Scale bars: 400 μm. Representative images of the transduced HFKs are shown in Supplementary Information Fig. S12. **c**, **d** Cumulative population doubling levels (PDLs) were calculated and plotted to days post-selection of HPECs (from prostate-1) and HFKs (foreskin-1) as indicated. **e** Early-passage (P2) KGM cultures of HPECs and HFKs were transduced and selected as above. Overexpression of Δ133p53α was confirmed by immunoblotting (lower band). Slightly slower migrating band represents their endogenous levels. GAPDH was a loading control. Representative images of the post-selected HPECs and HFKs are shown in Supplementary Information Fig. S13c, d. **f**, **g** The proliferation of all four cell lines was followed, beginning three passages after selection. Cumulative PDLs were plotted vs. time (days). The HPECs and HFKs shown in this figure were generated from prostate-1 and foreskin-1, respectively. Δ133p53α-overexpression experiments were performed in another set of HPECs (from prostate-2) and HFKs (from foreskin-2). Cumulative PDLs for proliferation assays are shown in Supplementary Information Fig. S15. Uncropped blot images are shown in Supplementary Information Figs. S32–33

Since primary epithelial cells undergo senescence after 10–11 passages in KGM culture (Fig. 2a, b) with loss of endogenous Δ133p53α levels (Figs. 1a and 2c), we overexpressed Δ133p53α in HPECs and HFKs at relatively late-passage (P6) by means of Δ133p53α lentiviral transduction (Fig. 4a). Δ133p53α overexpressing HPECs reproducibly bypassed the normal senescence barrier and continued to proliferate for six additional passages in KGM culture, whereas HPECs transduced with the empty vector stopped proliferating after two additional passages (Fig. 4b, c). A similar result was observed in Δ133p53α-overexpressing HFKs, which continued to proliferate for five additional passages in KGM compared to control cells (Fig. 4d and Supplementary Information Fig. S12).

To investigate the effect of Δ133p53α in the rapidly growing cells at early passages, we next overexpressed Δ133p53α in both HPEC and HFK cells at passage 2 (P2) and examined their proliferation status both short-term and long-term (Fig. 4e). In short-term assays, Δ133p53α-overexpressing HPECs became confluent after 7 days, whereas the control cells were growing slowly and reached only 40–45% (Supplementary Information Fig. S13a, b). In long-term assays, the empty vector control cells proliferated only 4–5 population doubling levels while the cells overexpressing Δ133p53α continued to grow an additional 15 population doubling levels (ten more passages) before they ceased proliferating and became senescent (Fig. 4f, g and Supplementary Information, Figs. S13c, d and S14). The extension of replicative lifespan by overexpressing Δ133p53α was also consistent in two other HPECs and HFKs generated from two different individuals (prostate-2 and foreskin-2) (Supplementary Information, Fig. S15). Therefore, we conclude that Δ133p53α overexpression promotes cell proliferation and can extend the replicative lifespan of primary HPECs and HFKs without the use of CR culture.

### Δ133p53α upregulates hTERT expression and telomerase activity in primary cells

**Fig. 5:**
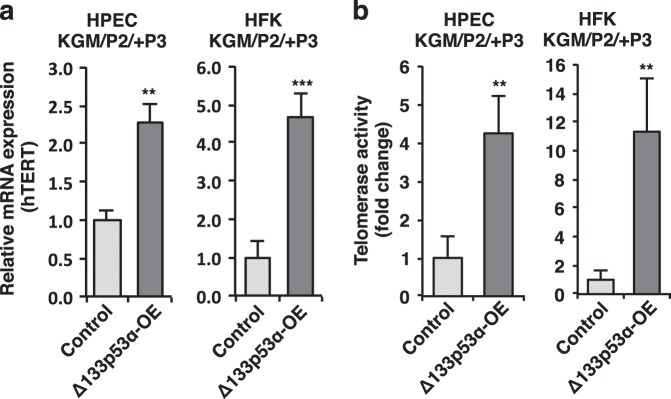
Δ133p53α increases hTERT mRNA levels and telomerase activity. **a** qRT-PCR analysis of hTERT mRNA levels in Δ133p53α-overexpressing (Δ133p53α-OE) HFK and HPEC cells three passages after selection in KGM. The empty GFP-tagged vector was used as a control. β2-microglobulin mRNA was used for normalization. Data are mean ± S.D. from three independent experiments. **P < 0.01; ***P < 0.001. **b** Telomerase activity was measured in the same Δ133p53α-OE HFK and HPEC cells three passages after selection in KGM. The empty GFP-tagged vector was used as a control. Data are expressed as mean ± S.D. from three independent experiments. **P < 0.01

In light of our current finding that Δ133p53α is highly expressed in CR cells and previous reports of increased hTERT mRNA and telomerase activity in CR cells^12,36^ we asked if there is a link between Δ133p53α and hTERT expression in primary epithelial cells. A 2–5-fold increase in hTERT mRNA expressions (compared to empty vector control cells) were observed in both the HPECs and HFKs overexpressing Δ133p53α (Fig. 5a). We also observed that overexpression of Δ133p53α had no effect on full-length p53 and E2F1, but reduced p53 targets p21, PUMA, NOXA, and BAX that are associated with cell-cycle arrest, apoptosis and senescence (Supplementary Information Fig. S16). Interestingly, the Δ133p53α-overexpressing epithelial cells also exhibited a 5–15-fold increase in telomerase activity (Fig. 5b). These results demonstrate that Δ133p53α upregulates hTERT expression in primary epithelial cells.

### hTERT is required for cell proliferation in Δ133p53α-overexpressing cells

**Fig. 6:**
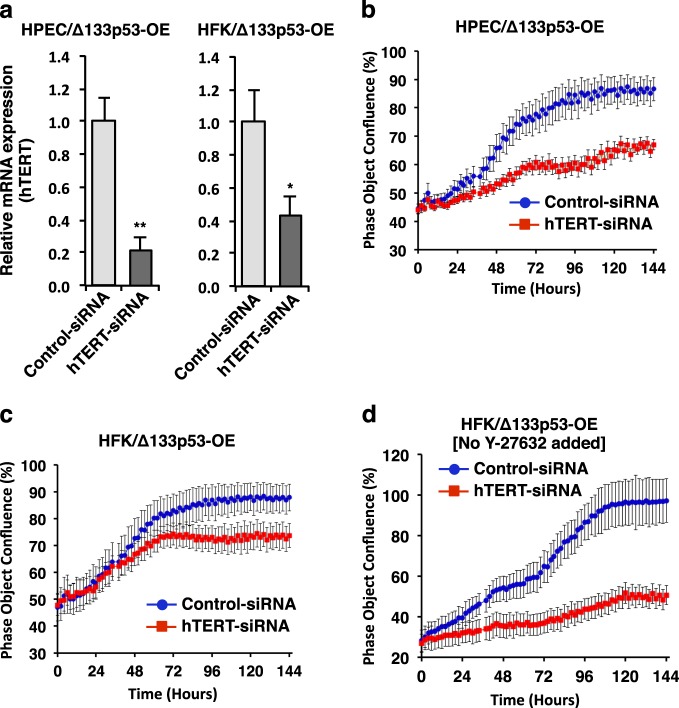
hTERT is required for cell proliferation in Δ133p53α-overexpressing cells. **a** KGM cultures of Δ133p53α-overexpressing (Δ133p53α-OE) HPECs (at passage 9) and HFKs (at passage 6) were transfected with hTERT-siRNA or control siRNA. hTERT mRNA expressions were measured by qRT-PCR. β2-microglobulin mRNA was used for normalization. Data are mean ± S.D. from three independent experiments. *P < 0.05; **P < 0.01. **b** Proliferation of Δ133p53α-OE HPECs and **c** HFKs were measured for 6 days after transfection in IncuCyte. Data are expressed as mean ± S.D. from triplicate wells. Representative images of HPECs and HFKs transfected with hTERT-siRNA and control siRNA are shown in Supplementary Information Fig. S17. **d** Proliferation of Δ133p53α-OE HFKs were measured in IncuCyte for 6 days after transfection with hTERT-siRNA or control siRNA in the absence of ROCK inhibitor Y-27632. Data are expressed as mean ± S.D. from triplicate wells

To further establish a link between Δ133p53α, hTERT and proliferation in primary cells, siRNA was used to knockdown endogenous hTERT in KGM cultures of Δ133p53α-overexpressing HFKs and HPECs. As shown (Fig. 6a), siRNA effectively reduced levels of hTERT mRNA by 60 (HFKs) and 80% (HPECs) compared to non-specific control siRNA. Interestingly, we observed clear inhibition of cell proliferation in both the cell types transfected with hTERT-siRNA, whereas cells transfected with control siRNA continued to proliferate at the same rate as none (no siRNA)-transfected cells (Fig. 6b, c and Supplementary Information Fig. S17a, b). Moreover, knockdown of hTERT expression in the Δ133p53α-overexpressing cells showed much stronger and immediate inhibition of cell proliferation when no ROCK inhibitor (Y-27632) was added to the culture medium (Fig. 6d). Altogether, these findings suggest that the induction of hTERT by Δ133p53α is required for continued cell proliferation.

### Δ133p53α immortalizes primary epithelial cells in cooperation with Rho-associated kinase (ROCK) inhibitor

**Fig. 7:**
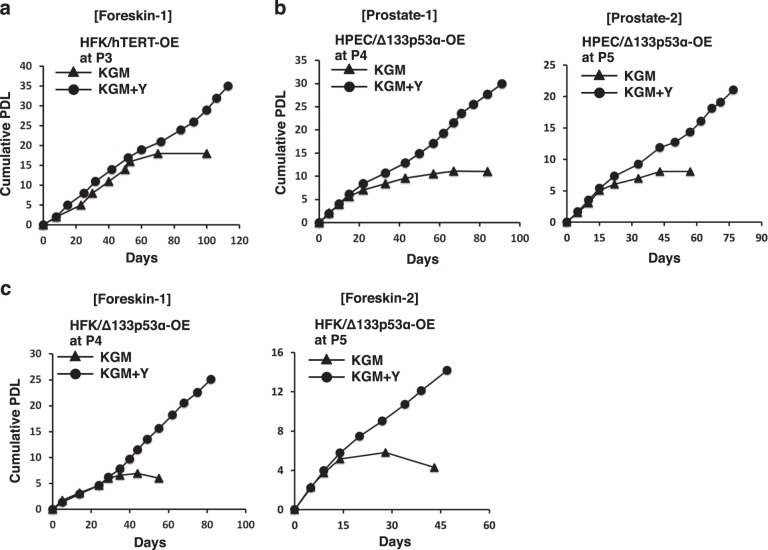
Δ133p53α immortalizes primary epithelial cells in cooperation with Y-27632. **a** hTERT-overexpressing HFKs (HFK/hTERT-OE) were cultured in KGM with or without Y-27632 beginning three passages after selection. Cumulative population doubling levels (PDLs) were plotted vs. time (days). The HFKs were generated from foreskin-1. **b** Δ133p53α-overexpressing HPECs (originated from prostate-1 and prostate-2), and **c** Δ133p53α-overexpressing HFKs (originated from foreskin-1 and foreskin-2) were cultured in KGM with or without Y-27632 beginning 4–5 passages after selection as indicated. Cumulative PDLs were plotted vs. time (days)

It is well documented that the ROCK inhibitor, Y-27632, enhances cell survival by inhibiting differentiation and apoptotic pathways^15,37,38^. Furthermore, the combined effects of ROCK inhibitor and increased hTERT expression are sufficient to conditionally immortalize primary epithelial cells^12,14,15^. Since we have shown that hTERT expression and telomerase activity are increased in Δ133p53α-overexpressing primary HFKs and HPECs, we asked if these cells could be immortalized by the addition of Y-27632. Initially, Y-27632 was not added to hTERT-overexpressing HFKs when culturing in KGM. HFKs overexpressing hTERT continued to proliferate indefinitely in presence of Y-27632, whereas the same cells stopped growing after seven passages without Y-27632 (Fig. 7a). To investigate if Δ133p53α can substitute for hTERT, Δ133p53α-overexpressing HFKs and HPECs were cultured in KGM in the presence or absence of Y-27632. As observed with hTERT overexpression, both HFKs- and HPECs-overexpressing Δ133p53α continued to grow indefinitely in KGM supplemented with Y-27632, whereas the same cells stopped growing after 6–8 passages without Y-27632 (Fig. 7b, c). Taken together, our data indicate that Δ133p53α overexpression and a ROCK inhibitor can immortalize primary epithelial cells and that the effect of Δ133p53α is related to its ability to induce hTERT expression.

## Discussion

In the current study, we show that the expression of both full-length p53 and its natural isoform, Δ133p53α, are upregulated in CR primary epithelial cells (HFKs and HPECs). In particular, Δ133p53α levels are closely correlated with proliferation. In non-CR cultures (KGM), early-passage primary cells express high levels of Δ133p53α, which decrease and eventually disappear as later-passage cells become senescent. Conversely, CR cultures of primary HFKs and HPECs exhibit rapid growth inhibition and loss of Δ133p53α expression when transferred to KGM. These findings are consistent with previous studies that demonstrated increased autophagic degradation of Δ133p53α in senescent primary human fibroblasts^25,39^. Moreover, we show that Δ133p53α overexpression consistently delays senescence with loss of p53 targets p21, PUMA, NOXA, and BAX that are implicated in cell-cycle arrest, apoptosis, or senescence (Supplementary Information Figs. S14 and S16) and extends the replicative lifespan of primary HFKs and HPECs, in agreement with the role of Δ133p53α dominant-negative inhibition of full-length p53 at p21 expression to suppress early onset of senescence^40^ and to enhance the generation of induced pluripotent stem cells^41^. The correlation between Δ133p53α expression and proliferation is further supported by the observation that siRNA knockdown of endogenous Δ133p53α under either synthetic serum-free medium (KGM) or CR culture results in immediate growth arrest. As previously reported, siRNA oligos used to knockdown Δ133p53 mRNA also reduce Δ160p53α protein isoform. Expression of Δ133p53α and Δ160p53α protein isoforms from the same Δ133p53 mRNA are cell type specific^24^. Therefore, it remained unclear whether any biological effects were associated with only Δ133p53 or Δ160p53 or both. We did not see any detectable Δ160p53α protein in western blots using CM1 antibody in the primary epithelial cells when Δ133p53α was either knocked-down (Supplementary Information Figs. S11 and S30) or overexpressed (Supplementary Information Fig. S31). Therefore, the possibility of Δ160p53α having any significant role in cell proliferation has been excluded in this study. Altogether, these results suggest that Δ133p53α plays an important role in the CR of primary epithelial cells.

hTERT, a specialized RNA-dependent DNA polymerase, maintains telomere length and plays a critical role in cellular survival and immortalization^42,43^. It is well known that wild-type p53 downregulates hTERT expression and that hTERT counteracts apoptosis induced by p53^44,45^. However, emerging details of p53-mediated cell survival^46,47^ and the regulation of telomere lengthening in cancer cells^48^ raise new questions regarding the interplay of p53 and hTERT. Recently, we showed that Δ133p53α physically interacts with wild-type p53 and likely inhibits p53-mediated apoptosis and senescence in a dominant-negative manner^41,49^ and speculated that increased Δ133p53α expression might be important for CR. In the present study, we show that the Δ133p53α-overexpressing primary epithelial cells exhibit increased levels of hTERT mRNA and telomerase activity (Fig. 5a, b), which is consistent with the loss of hTERT expression in the Δ133p53α-siRNA-knockdown cells (Fig. 3f). In addition, we observed rapid cell death when siRNA was used to knockdown endogenous hTERT in the Δ133p53α-induced reprogrammed cells (Fig. 6b–d). This hTERT-siRNA-knockdown effect on growth inhibition was much stronger and immediate in absence of ROCK inhibitor (Fig. 6d). These findings show that hTERT expression and telomerase activity are required for long-term passaging or CR of epithelial cells, and that Δ133p53α-mediated upregulation of hTERT contributes to inhibit full-length p53-mediated apoptosis or cellular senescence in vitro^50^ (Supplementary Information Fig. S16).

hTERT is essential for maintaining or elongating telomeres, thus allowing for continued cell replication^51^. However, our  previous studies showed that HPV E6-induced telomerase activity was dissociated from telomere maintenance during cell immortalization^52^. This is consistent with our findings that CR and Δ133p53α overexpression result in long-term cell proliferation despite the erosion and shortening of telomere length. The mechanisms that stabilize these short telomeres and prevent senescence or apoptosis is incompletely understood at this time. What is clear is that CR culture conditions and Δ133p53α overexpression both appear to function as inducers of hTERT and that this activity, when combined with the cytoskeletal destabilizing activity of ROCK inhibitor, is critical for the prolonged proliferation of “primary” cell cultures.

## Electronic supplementary material

Supplementary information
