# Supplementary material for: Δ133p53α, a natural p53 isoform, contributes to conditional reprogramming and long-term proliferation of primary epithelial cells
Source: Cell Death Dis. 2018 Jul 3;9(7):750. doi: 10.1038/s41419-018-0767-7 (PMC6030220; doi:10.1038/s41419-018-0767-7)
Supplement: Supplementary file 1 — Supplementary information [file 41419_2018_767_MOESM1_ESM.pdf]

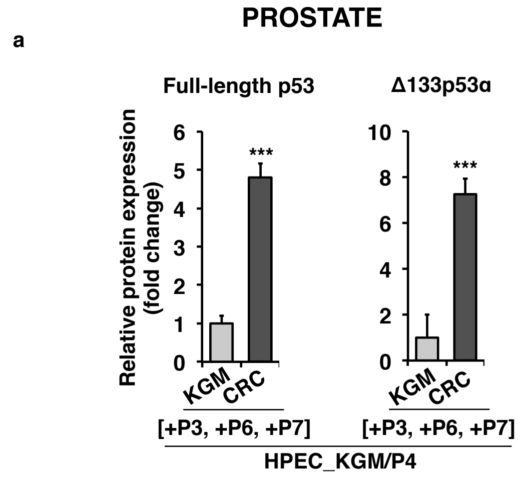

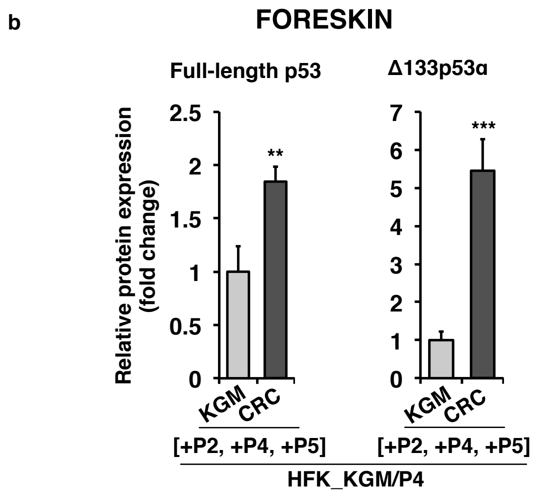

Figure S1 Increased expression of full-length p53 and Δ133p53α in CR cells. (a) Bar graph of the immunoblot analysis of HPECs shown in Fig. 1a and (b) HFKs shown in Fig. 1b. Data are mean ± S.D. from 3 independent biological samples. $**P < 0.01$; $***P < 0.001$.

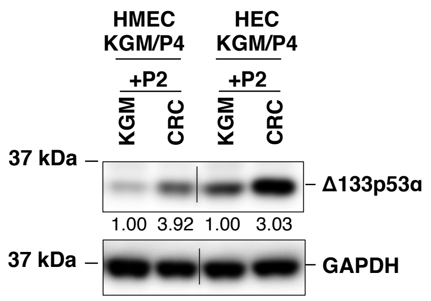

Figure S2 Immunoblot analysis of Δ133p53α protein in HMEC and HFK cells in KGM or CRC. GAPDH was a loading control. Normalized densitometric values are indicated below each lane relative to +P2 KGM cells (defined as 1.0).

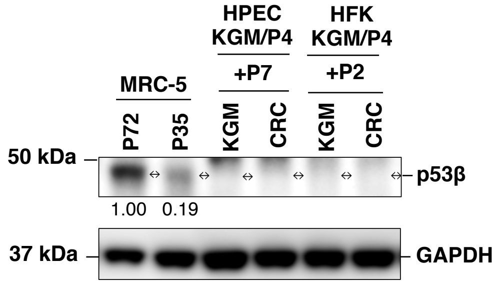

Figure S3 Immunoblot analysis of p53β expression in HPECs and HFKs in KGM or CRC. Late-passage (P72) and early-passage (P35) of human fibroblast MRC-5 were used as positive and negative controls, respectively. GAPDH was a loading control. Expression levels of p53β are indicated below each lane, wherever detected. (The level in MRC-5 (P72) is defined as 1.0). p53β was undetectable in primary epithelial cells.

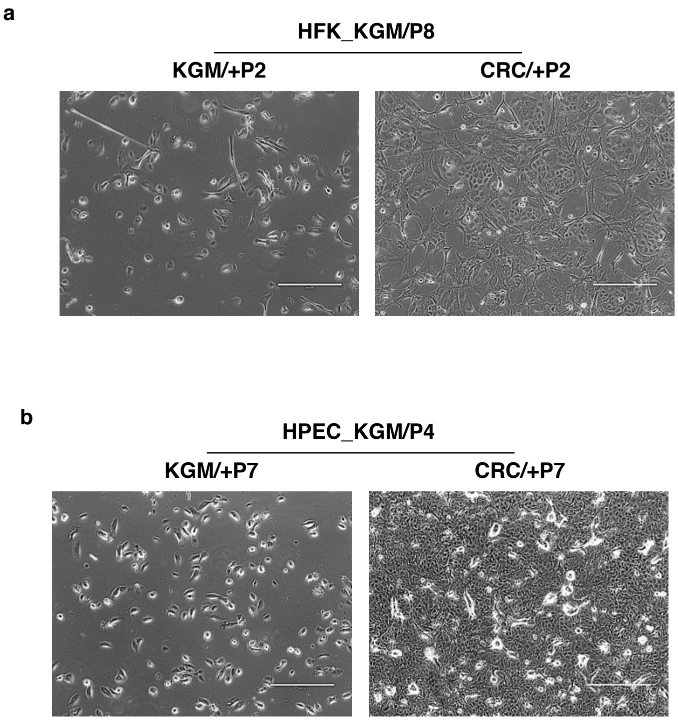

Figure S4 CRC induces proliferation of late-passage KGM cultures of primary epithelial cells. (**a**) Passage 8 HFKs were switched from KGM to CRC and cultured for 2 more passages (+P2). (**b**) Passage 4 HPECs were switched from KGM to CRC and cultured for 7 more passages (+P7). Scale bars: 400 µm.

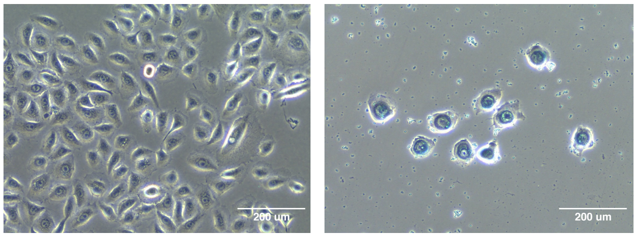

Figure S5 Representative images of senescence-associated β-galactosidase staining of HFKs cultured in KGM at early passage (P4) or late passage (P10). Scale bars: 200 µm.

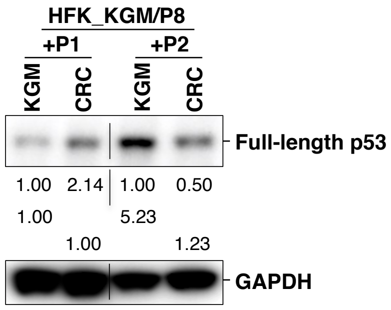

Figure S5 Immunoblot analysis of full-length p53 in the late-passage (KGM/P8) HFKs cultured in KGM or CRC for additional 2 passages (+P1, +P2). GAPDH was a loading control.

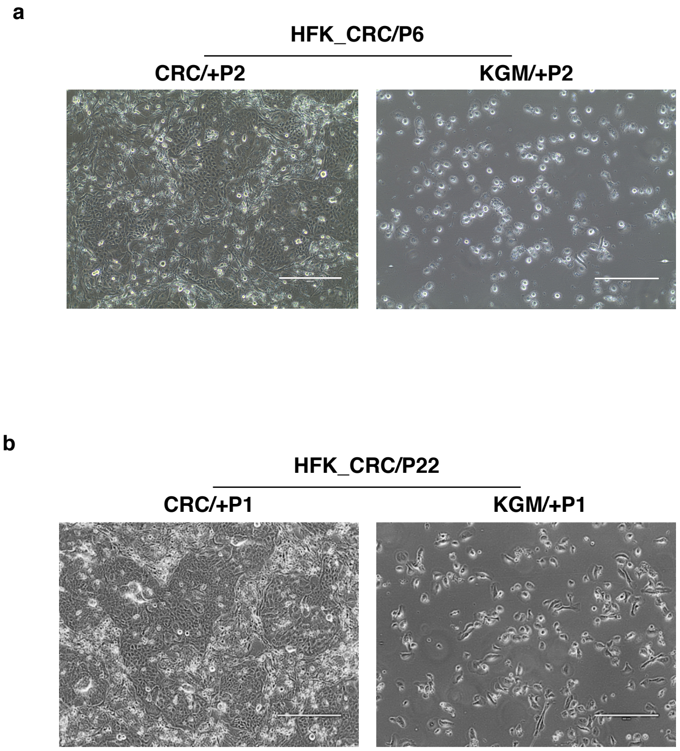

Figure S7 Transition from CRC to KGM culture rapidly decreases proliferation. (**a**) Passage 6 HFKs in CRC were switched to KGM for 2 more passages (+P2). (**b**) Passage 22 HFKs in CRC were switched to KGM for 1 more passage (+P1). Representative images are shown. Scale bars: 400 µm.

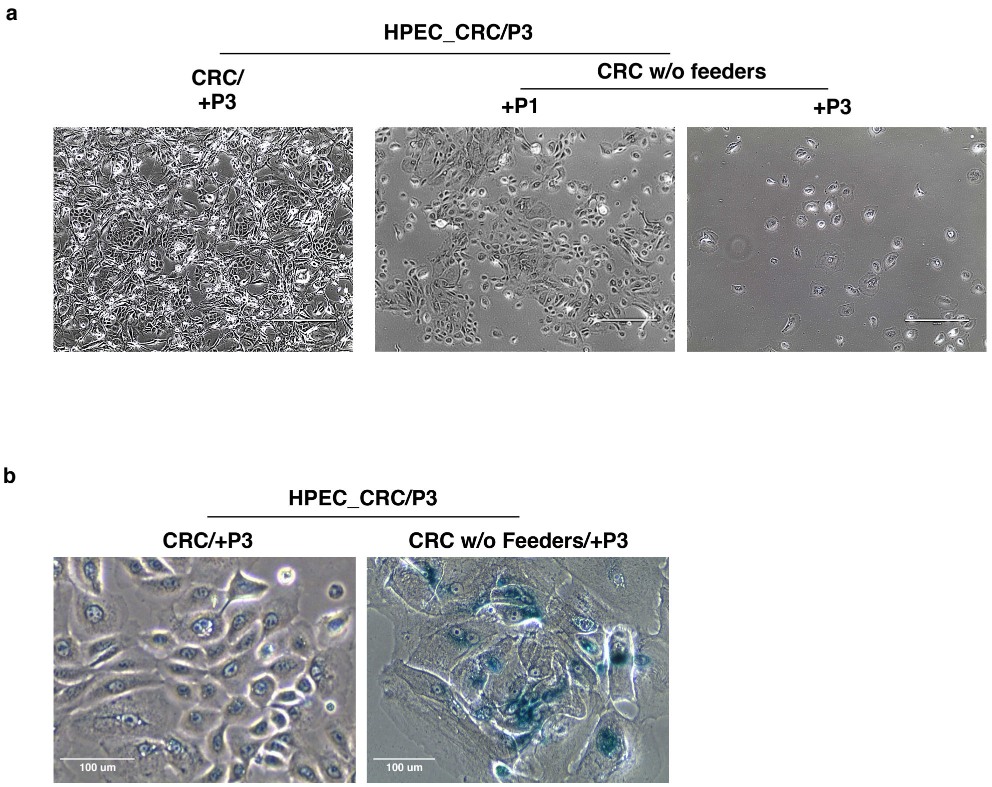

Figure S8 Irradiated feeder fibroblasts are essential for epithelial cell proliferation in CRC. (**a**) Irradiated feeder cells were withdrawn from rapidly proliferating passage 3 HPECs in CRC (HPEC_CRC/P3). The HPECs were then cultured without feeders in CRC for 1-3 additional passages (+P1, +P3). As a control, the HPECs also were passaged 3 times in complete CRC. Scale bars: 400 µm. (**b**) Representative images for senescence-associated β-galactosidase staining of HPECs cultured for 3 passages in CRC with or without feeders. Scale bars: 100 µm.

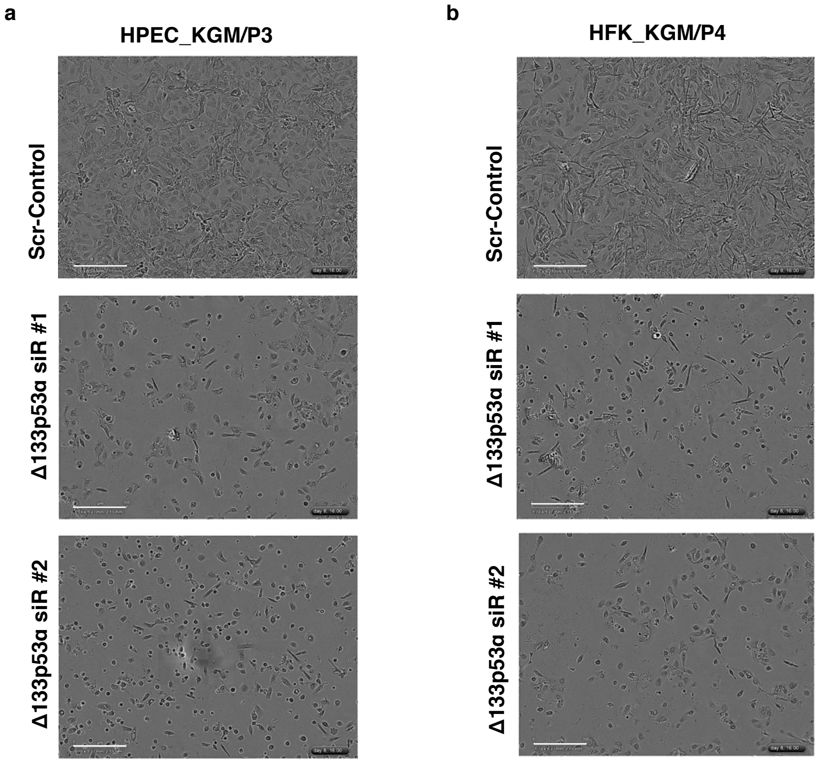

Figure S9 Knockdown of endogenous Δ133p53α inhibits cell proliferation. (**a**) Early-passage KGM cultures of HPECs (P3) and (**b**) HFKs (P4) were transfected with scrambled siRNA (Scr-Control), Δ133p53α-siR#1 or Δ133p53α-siR#2 and photographed 8 days after transfection. Scale bars: 300 µm.

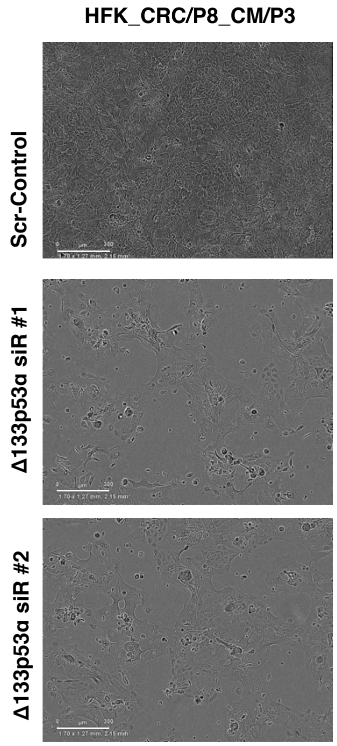

Figure S10 Knockdown of endogenous Δ133p53α inhibits cell proliferation. HFKs grown in CR conditions for 11 passages (CRC/P8 + CM/P3) were transfected with Scr-Control siRNA, Δ133p53α siR#1 or Δ133p53α siR#2. Representative images at 7 days post-transfection are shown. Scale bars: 300 µm.

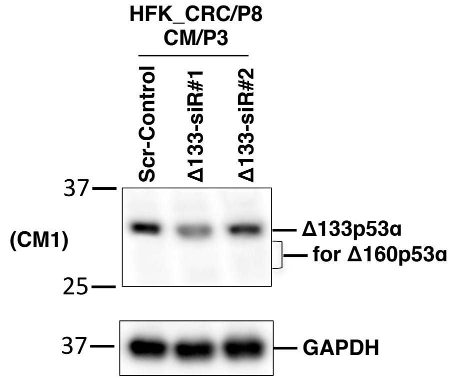

Figure S11 Immunoblot analysis of Δ133p53α and Δ160p53α protein isoforms using CM1 antibody. HFKs grown in CR conditions for 11 passages (CRC/P8 + CM/P3) were transfected with Scr-Control siRNA, Δ133p53α siR#1 or Δ133p53α siR#2. GAPDH was a loading control. Δ160p53α protein expression was undetectable in primary epithelial cells.

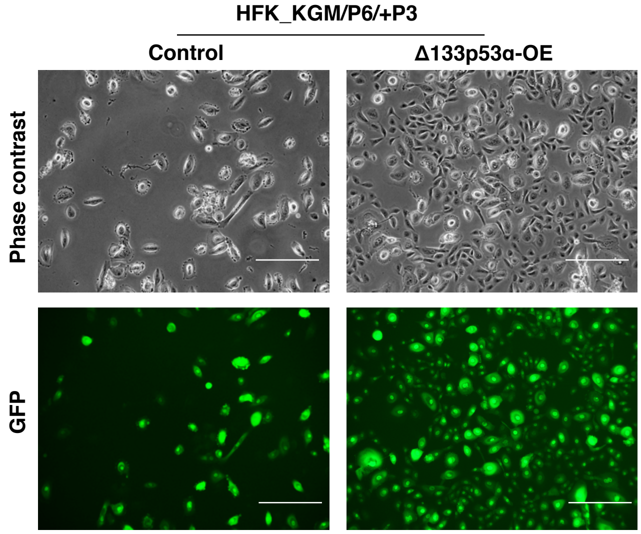

Figure S12 Overexpression of Δ133p53α promotes proliferation and extends the replicative lifespan of primary epithelial cells. A late-passage (P6) KGM culture of HFKs was transduced with lentivirus containing GFP-tagged empty vector (Control) or GFP-tagged Δ133p53α (Δ133p53α-OE). Representative images of the Control and Δ133p53α-OE HFKs are shown 3 passages after selection (HFK_KGM/P6/+P3). Scale bars: 400 μm.

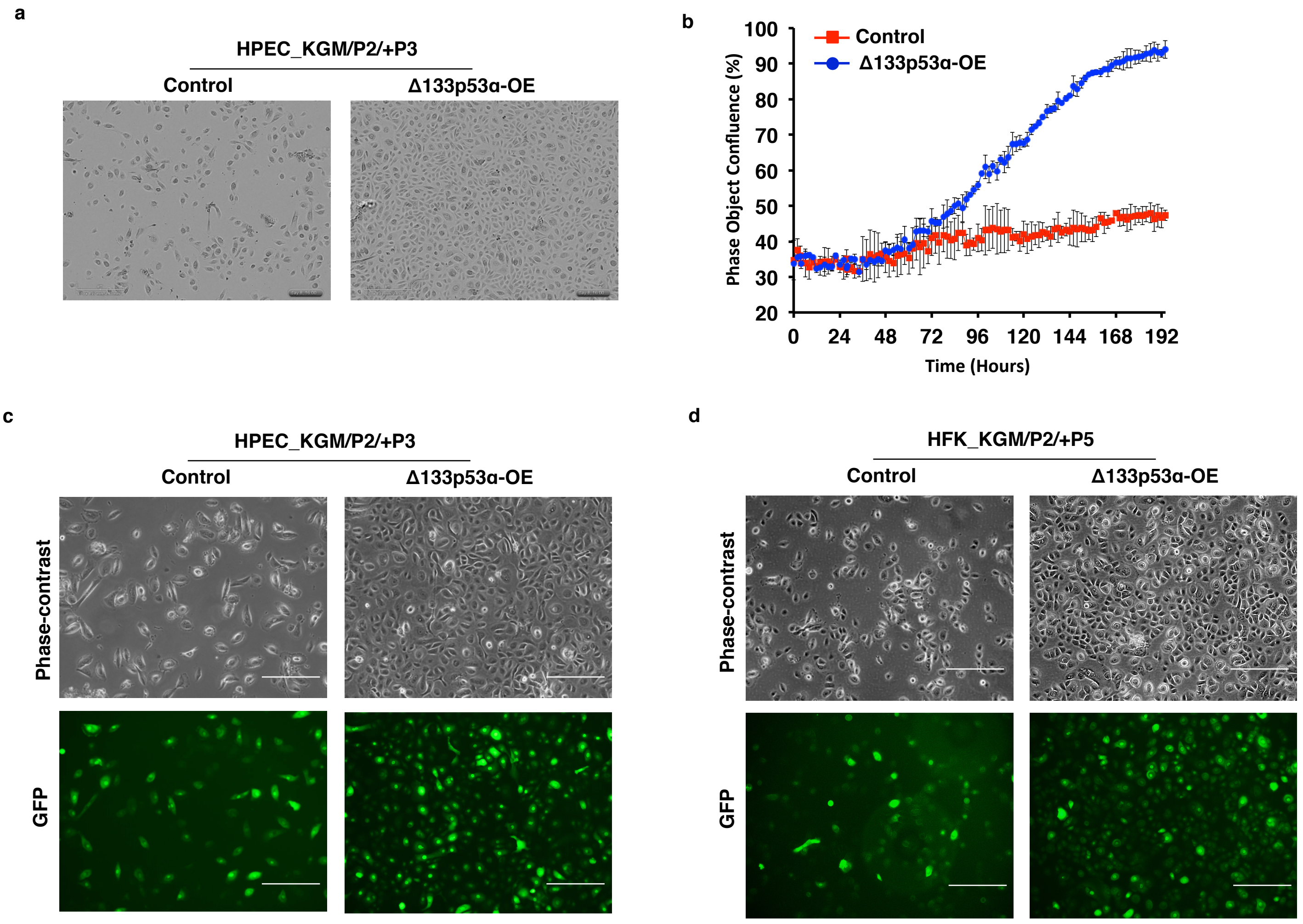

Figure S13 Overexpression of Δ133p53α promotes proliferation of early passaged epithelial cells. (**a**) KGM passage 2 of HPECs was transduced with lentivirus containing GFP-tagged empty vector (Control) or GFP-tagged Δ133p53α (Δ133p53α-OE). Representative IncuCyte images are shown at passage 3 after selection (HPEC_KGM/P2/+P3). Scale bars: 300 µm. (**b**) Cell proliferation assay by IncuCyte. Phase object confluency were measured for 8 days in the Control or in Δ133p53α-OE cells at passage 3 post-selection. Data are the mean ± s.d. from triplicate wells. (**c, d**) Representative images of the Control or Δ133p53α-OE HPEC cells (c) and HFK cells (d) at passage 3 (+P3) or passage 5 (+P5) post-selection, respectively. Scale bars: 400 µm.

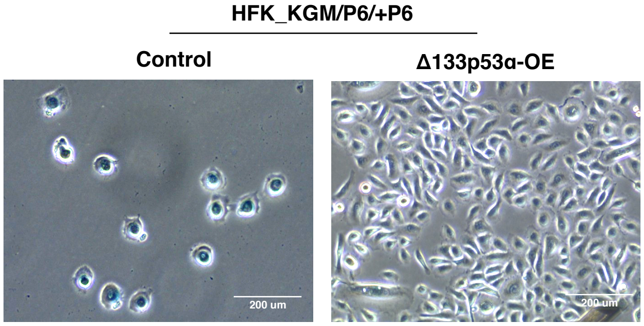

Figure S14 Representative images of senescence-associated β-galactosidase staining of transduced HFKs with lentivirus containing GFP-tagged empty vector (Control) or GFP-tagged Δ133p53α (Δ133p53α-OE). Selected cells were grown for 6 more passages in KGM before staining. Scale bars: 200 µm.

a

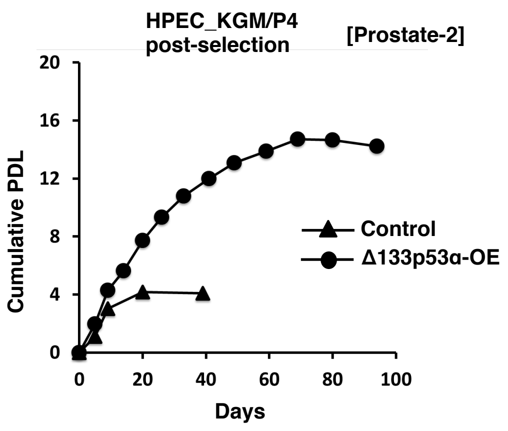

b

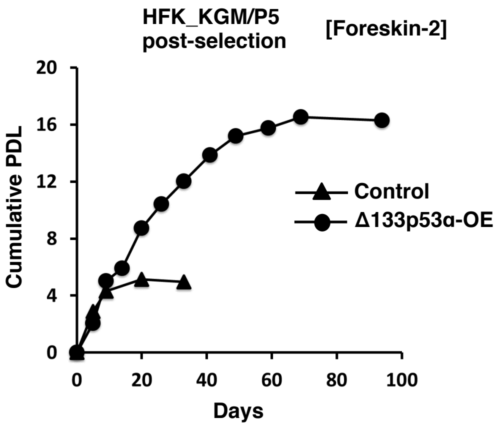

Figure S15 Overexpression of Δ133p53α extends replicative lifespan of normal epithelial cells. HPECs (from prostate-2) in KGM at passage 4 and HFKs (from foreskin-2) in KGM at passage 5 were transduced with lentivirus containing GFP-tagged empty vector (Control) or GFP-tagged Δ133p53α (Δ133p53α-OE). Cumulative population doubling levels (PDLs) were calculated and plotted to days post-selection of HPECs (**a**) and HFKs (**b**) as indicated.

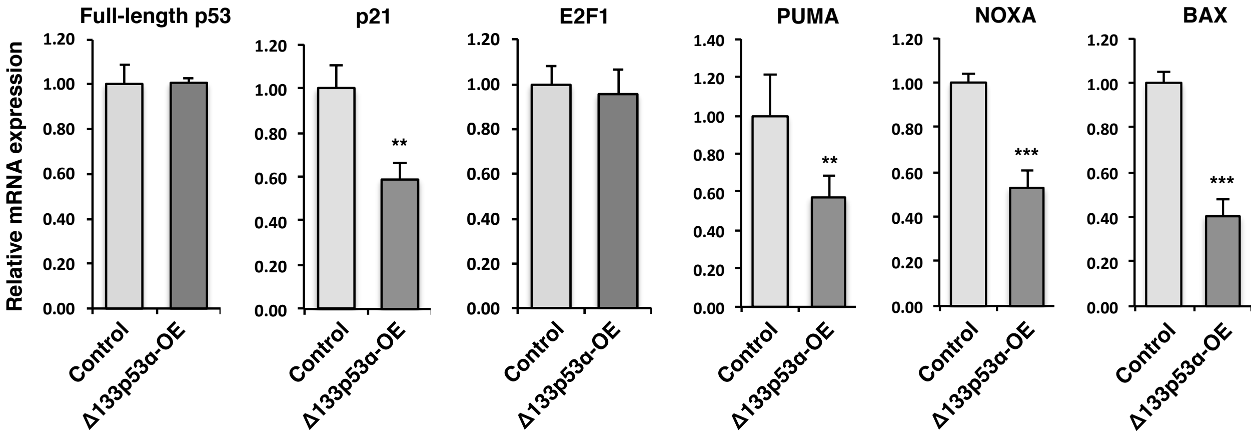

Figure S16 Expression of full-length p53, p21, E2F1, PUMA, Noxa and Bax mRNA in the HFK cells transduced with empty vector (Control) or Δ133p53α (Δ133p53α-OE) were measured by qRT-PCR. β2-microglobulin mRNA was used for normalization. Data are mean ± S.D. from 3 independent experiments. ***$P < 0.01$*, ****$P < 0.001$*.

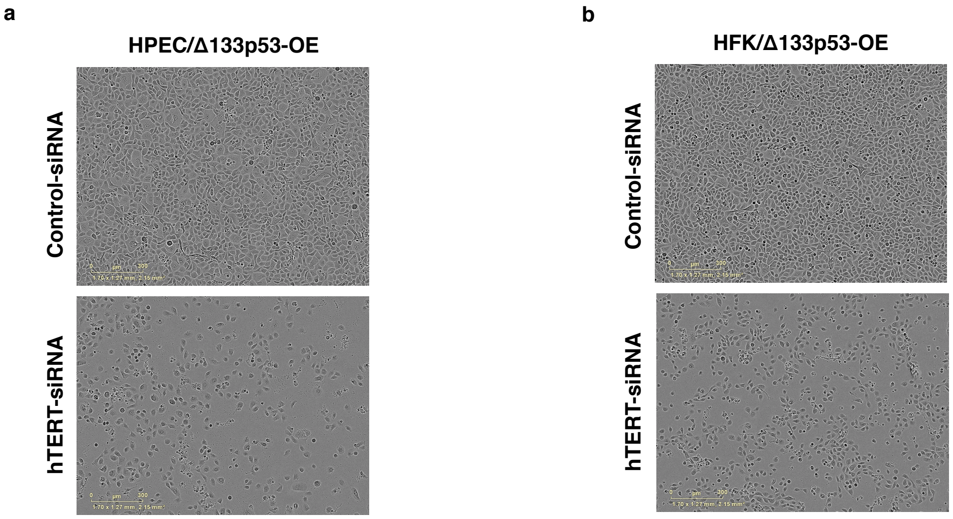

Figure S17 Knockdown of endogenous hTERT inhibits proliferation in Δ133p53α-overexpressing primary epithelial cells. (**a**) Antibiotic selected Δ133p53α-OE HPECs (at passage 9 in KGM) and (**b**) HFKs (at passage 6 in KGM) were transiently transfected with negative control siRNA or hTERT siRNA. Representative IncuCyte images are shown 5 days post-transfection. Scale bars: 300 µm.

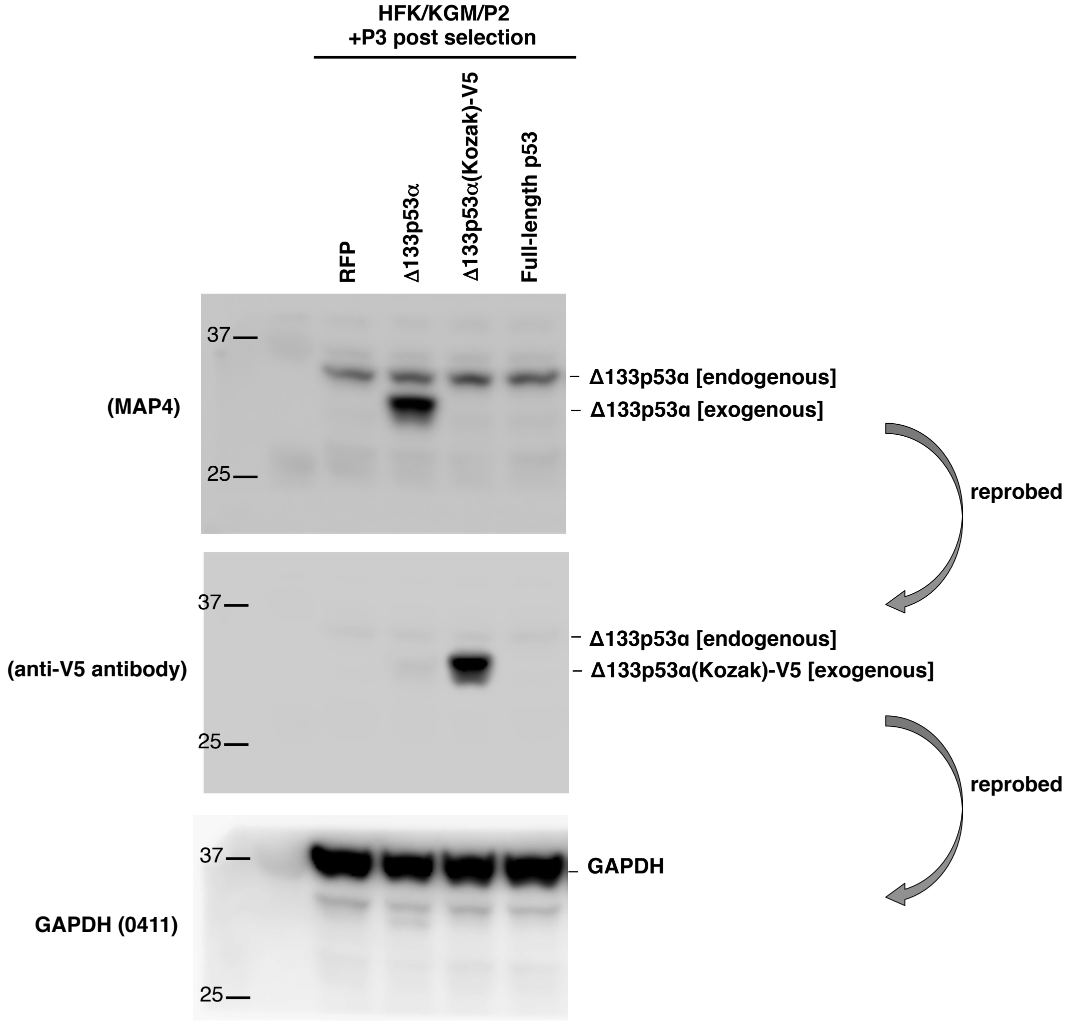

Figure S18 Specificity of MAP4 antibody for Δ133p53α protein isoform. HFK cells transduced with lentivirus containing RFP-control, Δ133p53α, Δ133p53α(Kozak)-V5 or full-length p53 (p53-FL) were used for immunoblotting. (***upper blot***) Immunoblot analysis of Δ133p53α (endogenous and exogenous) using MAP4 antibody. Both the Δ133p53α forms are indicated. The reactivity was lost upon N-terminal modification [Kozak modification (MGFCQLAKTC….), inserted amino acid is underlined; Kozak M. Nucleic Acids Res. 1987, 15:8125-8148] and C-terminal V5-tag of Δ133p53α in the Δ133p53α(Kozak)-V5 lane. (***middle blot***) Immunoblot analysis using anti-V5 antibody that detected only Δ133p53α(Kozak)-V5. (***lower blot***) GAPDH was used as a loading control.

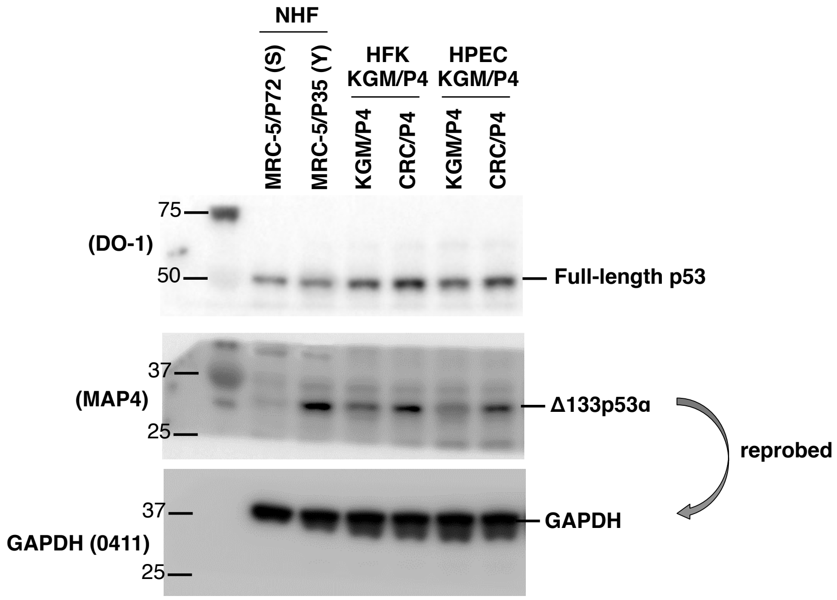

Figure S19 Immunoblot analysis to confirm Δ133p53α detection using MAP4 antibody. HFK and HPEC cells cultured in KGM or CRC were run in parallel to normal human fibroblast (MRC-5) at passage 35 (Y, young) and at passage 72 (S, senescent) as a positive and negative control, respectively, for Δ133p53α in the same blot. Full-length p53 expression was consistent as shown in this manuscript and in our previous reports (Fujita et al, Nat. Cell Biol. 2009; Mondal et al, J. Clin. Investig. 2013). GAPDH was used as a loading control.

For Fig. 1a

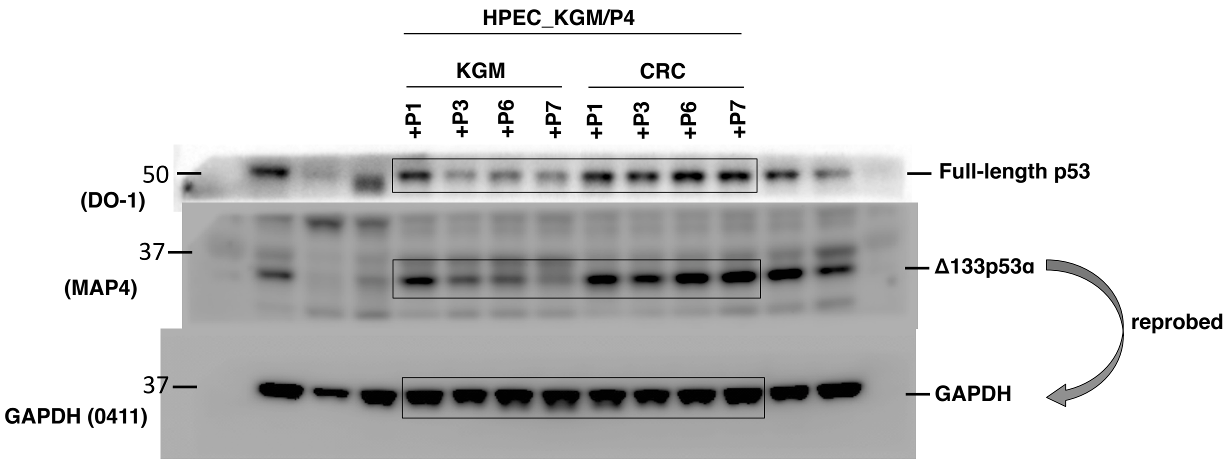

Figure S20 Original scans of immunoblots. The rectangular areas of the blots were put together and shown in the figure mentioned above.

For Fig. 1b (left panel)

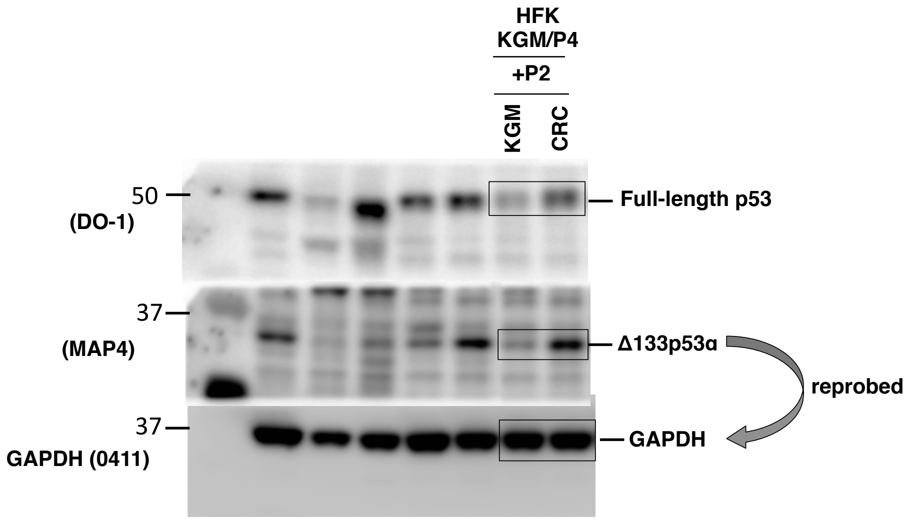

Figure S21 Original scans of immunoblots. The rectangular areas of the blots were put together and shown in the figure mentioned above.

For Fig. 1b (middle panel)

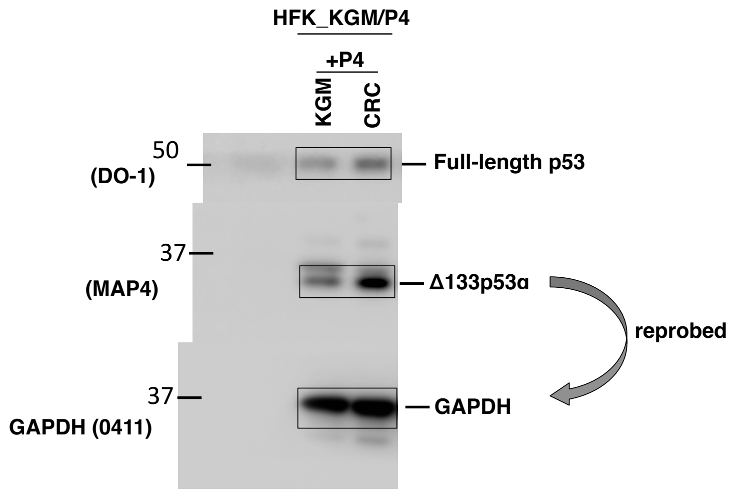

Figure S22 Original scans of immunoblots. The rectangular areas of the blots were put together and shown in the figure mentioned above.

For Fig. 1b (right panel)

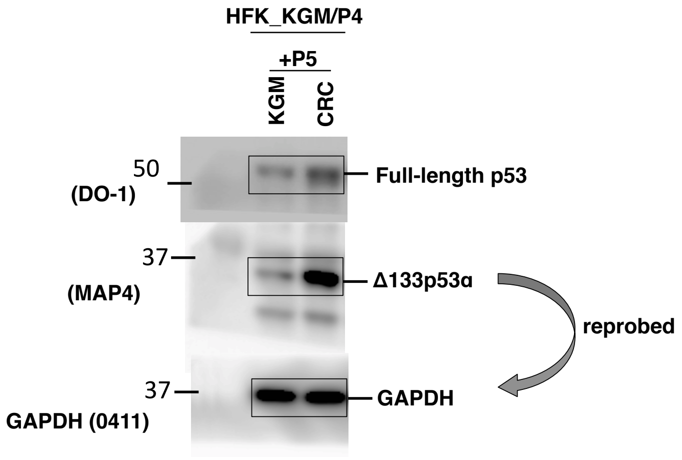

Figure S23 Original scans of immunoblots. The rectangular areas of the blots were put together and shown in the figure mentioned above.

For Fig. S2

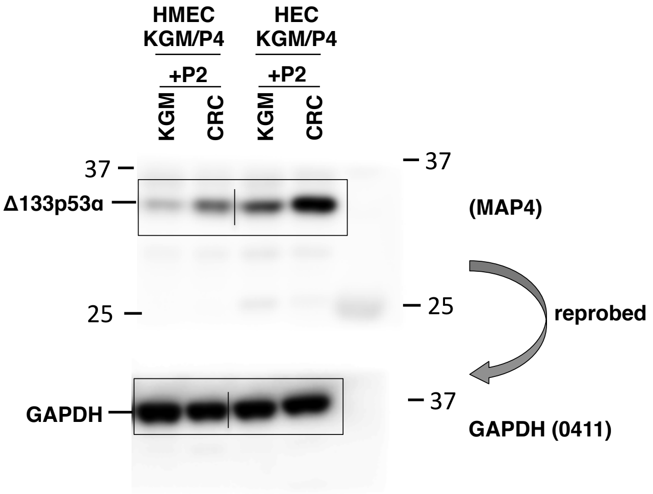

Figure S24 Original scans of immunoblots. The rectangular areas of the blots were put together and shown in the figure mentioned above.

For Fig. S3

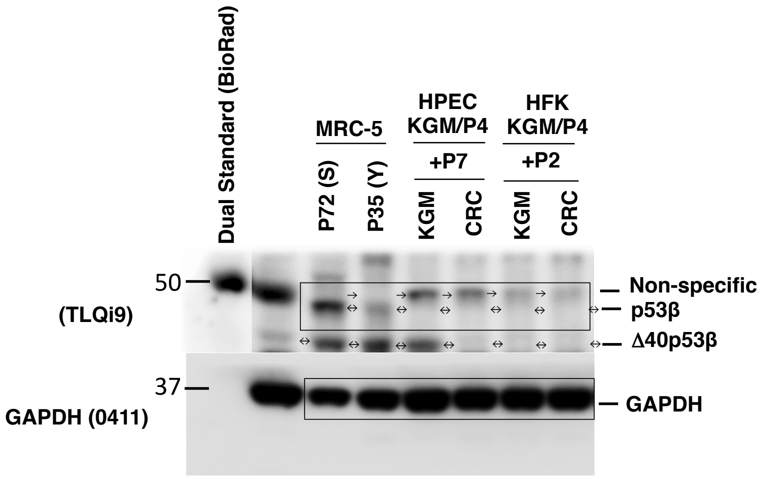

Figure S25 Original scans of immunoblots. The rectangular areas of the blots were put together and shown in the figure mentioned above.

For Fig. 2c and Fig. S5

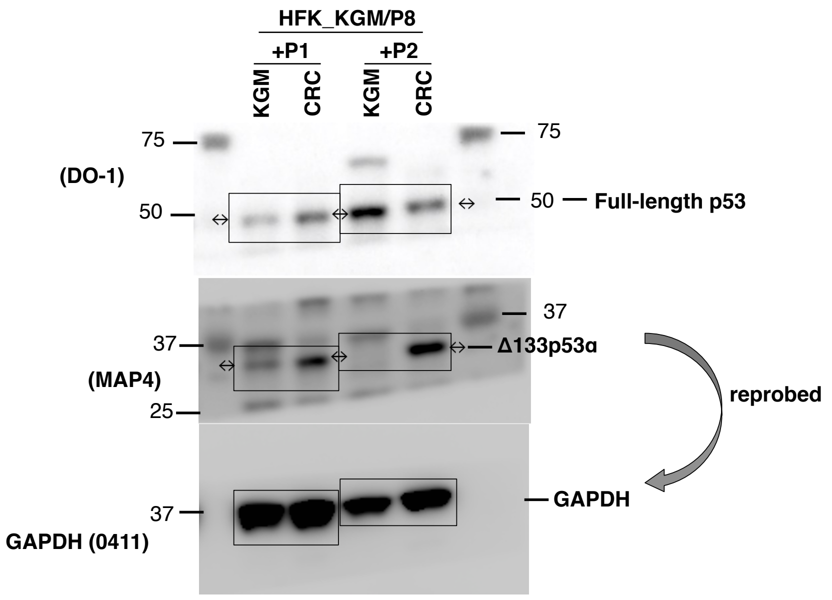

Figure S26 Original scans of immunoblots. The rectangular areas of the blots were put together and shown in the figures mentioned above.

For Fig. 2e

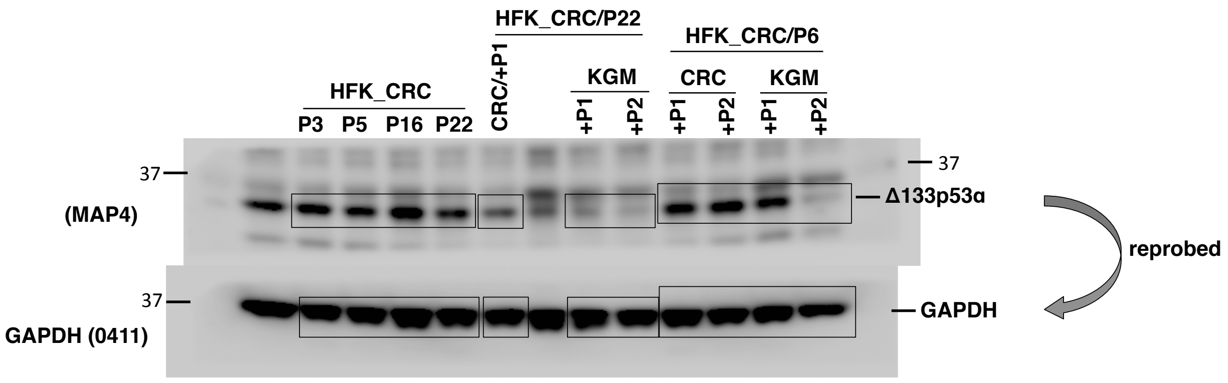

Figure S27 Original scans of immunoblots. The rectangular areas of the blots were put together and shown in the figure mentioned above.

For Fig. 2g

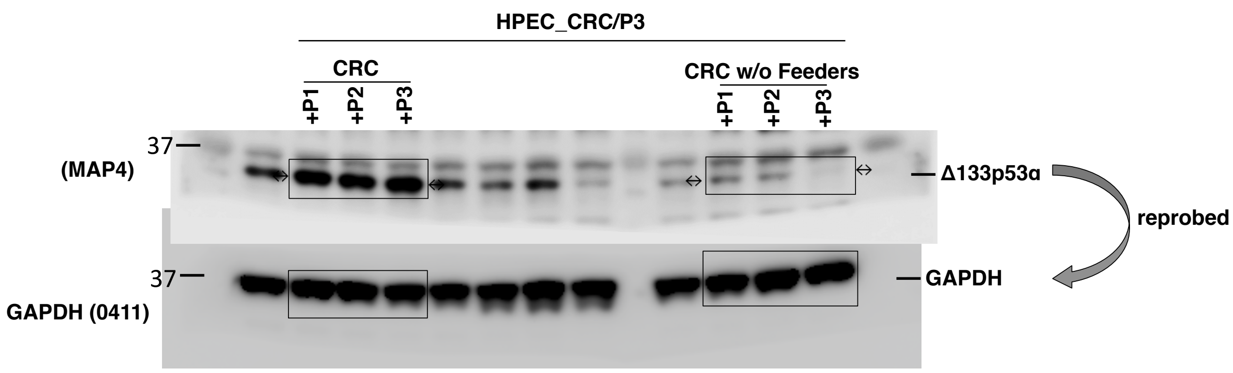

Figure S28 Original scans of immunoblots. The rectangular areas of the blots were put together and shown in the figure mentioned above.

For Fig. 3a (left panel)

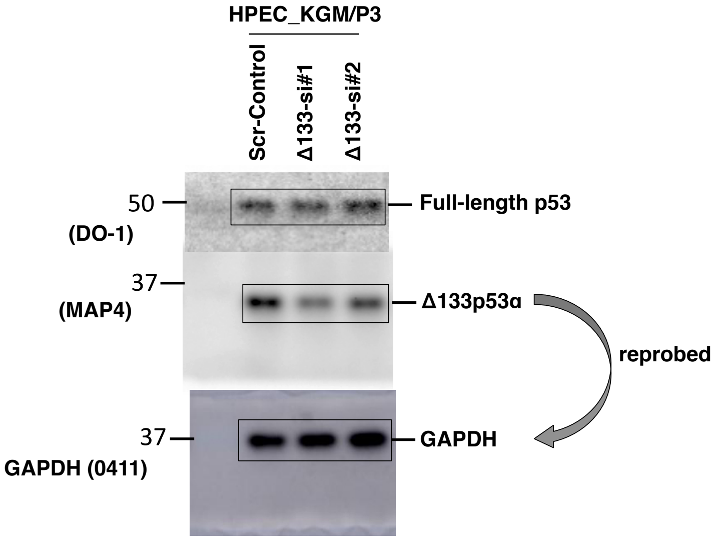

For Fig. 3a (right panel)

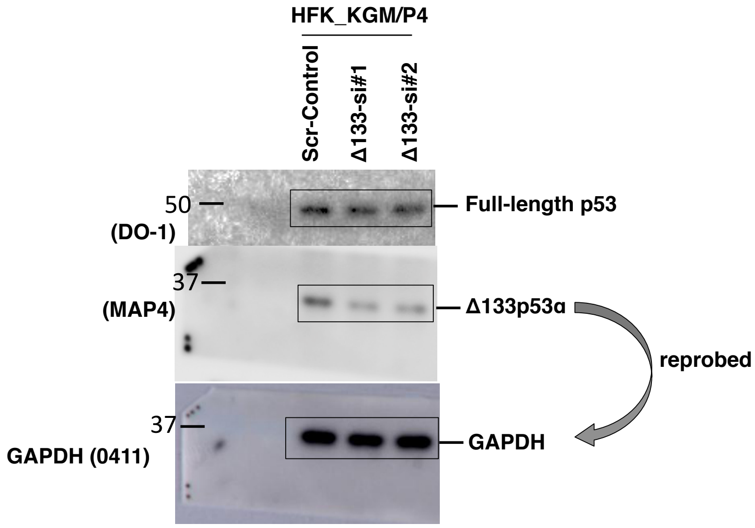

Figure S29 Original scans of immunoblots. The rectangular areas of the blots were put together and shown in the figures mentioned above.

For Fig. 3d and S11

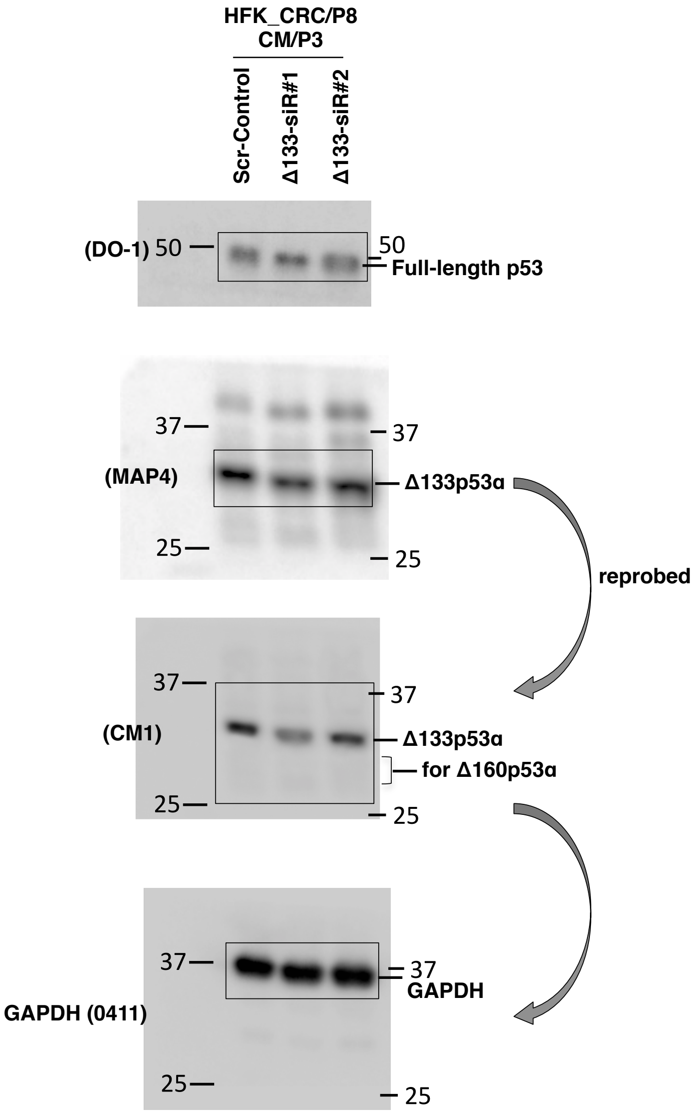

Figure S30 Original scans of immunoblots. The rectangular areas of the blots were put together and shown in the figure mentioned above. Samples were run on 10% SDS-PAGE.

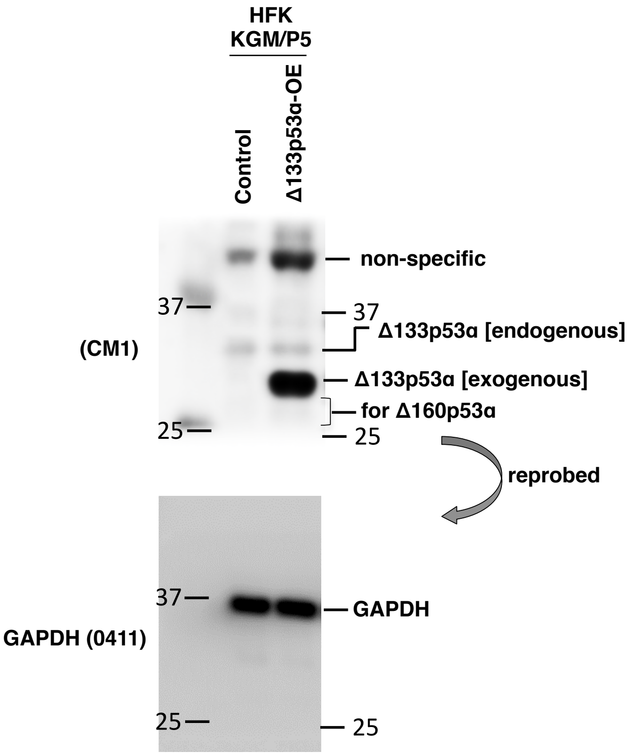

Figure S31 Immunoblot analysis of Δ133p53α and Δ160p53α protein isoforms using CM1 antibody in Δ133p53α-overexpressing HFKs. GAPDH was a loading control. Δ160p53α protein expression was undetectable when Δ133p53α was expressed abundantly. Samples were run on 10% SDS-PAGE.

For Fig. 4a
(left panel)

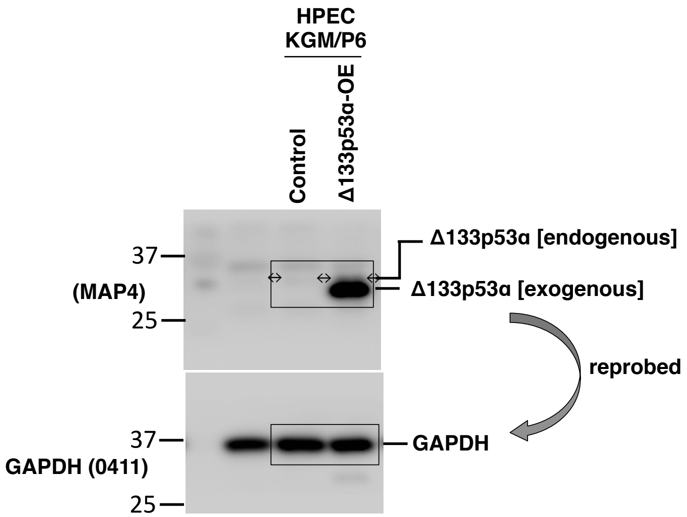

For Fig. 4a
(right panel)

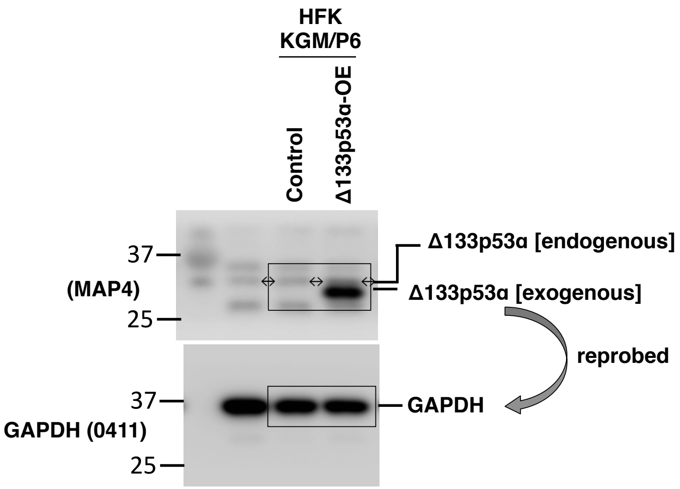

Figure S32 Original scans of immunoblots. The rectangular areas of the blots were put together and shown in the figure mentioned above. Samples were run on 14% SDS-PAGE.

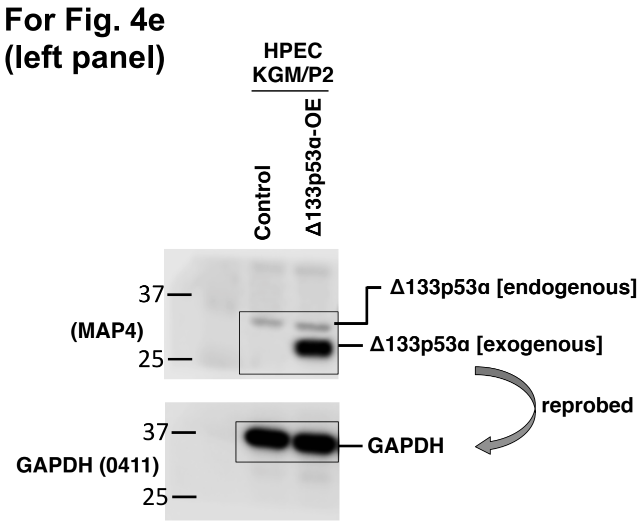

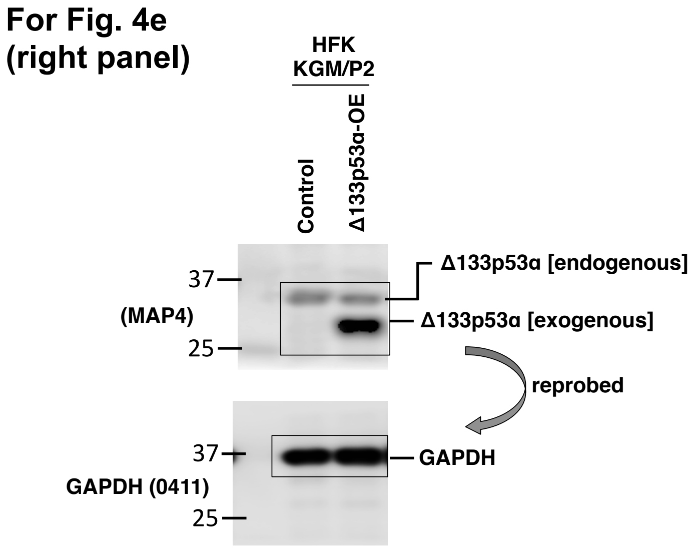

Figure S33 Original scans of immunoblots. The rectangular areas of the blots were put together and shown in the figure mentioned above. Samples were run on 12% SDS-PAGE.
